# CHIP-mediated ubiquitin degradation of BCAT1 regulates glioma cell proliferation and temozolomide sensitivity

**DOI:** 10.1038/s41419-024-06938-6

**Published:** 2024-07-29

**Authors:** Zhuo Lu, Xiao-Yu Wang, Kai-Yi He, Xin-Hao Han, Xing Wang, Zhen Zhang, Xin-Hui Qu, Zhi-Ping Chen, Xiao-Jian Han, Tao Wang

**Affiliations:** 1 Department of Thoracic Surgery, The First Affiliated Hospital, Jiangxi Medical College, Nanchang University, Nanchang, Jiangxi 330006 P.R. China; 2 Institute of Geriatrics, Jiangxi Provincial People’s Hospital, The First Affiliated Hospital of Nanchang Medical College, Nanchang, Jiangxi 330006 P.R. China; 3 Centre for Medical Research and Translation, Jiangxi Provincial People’s Hospital, The First Affiliated Hospital of Nanchang Medical College, Nanchang, Jiangxi 330006 P.R. China; 4 Institute of Clinical Medicine, Jiangxi Provincial People’s Hospital, The First Affiliated Hospital of Nanchang Medical College, Nanchang, Jiangxi 330006 P.R. China; 5 The Second Department of Neurology, Jiangxi Provincial People’s Hospital, The First Affiliated Hospital of Nanchang Medical College, Nanchang, Jiangxi 330006 P.R. China

**Keywords:** CNS cancer, Tumour-suppressor proteins, Ubiquitylation, Cell death

## Abstract

Glioma, a malignant and infiltrative neoplasm of the central nervous system, poses a significant threat due to its high mortality rates. Branched-chain amino acid transaminase 1 (BCAT1), a key enzyme in branched-chain amino acid (BCAA) catabolism, exhibits elevated expression in gliomas and correlates strongly with poor prognosis. Nonetheless, the regulatory mechanisms underlying this increased BCAT1 expression remains incompletely understood. In this study, we reveal that ubiquitination at Lys360 facilitates BCAT1 degradation, with low ubiquitination levels contributing to high BCAT1 expression in glioma cells. The Carboxyl terminus of Hsc70-interacting protein (CHIP), an E3 ubiquitin ligase, interacts with BCAT1 via its coiled-coil (CC) domain, promoting its K48-linkage ubiquitin degradation through proteasomal pathway. Moreover, CHIP-mediated BCAT1 degradation induces metabolic reprogramming, and impedes glioma cell proliferation and tumor growth both in vitro and in vivo. Furthermore, a positive correlation is observed between low CHIP expression, elevated BCAT1 levels, and unfavorable prognosis among glioma patients. Additionally, we show that the CHIP/BCAT1 axis enhances glioma sensitivity to temozolomide by reducing glutathione (GSH) synthesis and increasing oxidative stress. These findings underscore the critical role of CHIP/BCAT1 axis in glioma cell proliferation and temozolomide sensitivity, highlighting its potential as a diagnostic marker and therapeutic target in glioma treatment.

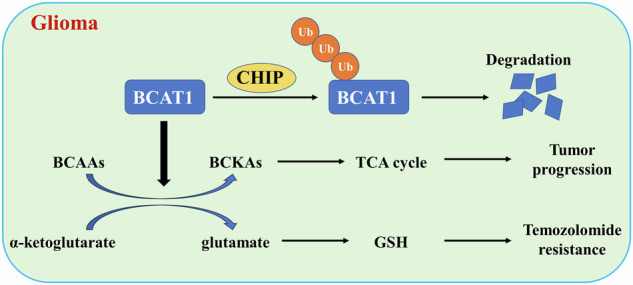

## Introduction

Glioma, a class of malignant brain tumors originating from glial cells, poses a multifaceted challenge in medical research and patient management [1]. Characterized by a diverse array of signs and symptoms, including persistent headaches, seizures, and neurological deficits, early diagnosis of glioma remains problematic [2]. The etiology of glioma is complex, involving genetic mutations, environmental factors, viral infections and immune system dysfunction [3]. With a grim prognosis, especially for high-grade glioblastomas, the aggressive and infiltrative nature of these tumors necessitate a multidisciplinary approach to treatment. Surgical resection, radiation therapy, and chemotherapy, including the use of temozolomide, constitute the backbone of current therapeutic strategies [4]. However, the infiltrative nature of gliomas often leaves microscopic remnants, and the emergence of temozolomide resistance stands as a significant challenge in chemotherapy for glioma [5]. Given the persistently low median survival rates for glioma patients [6], it is imperative to gain new insights into the complexities of glioma, with a particular emphasis on overcoming temozolomide resistance, to improve prognosis and enhance the quality of life for glioma patients.

Increased BCAA metabolism is a characteristic feature of glioma [7]. BCAA metabolism encompasses two distinct pathways with divergent end products: one yields acyl-CoA derivatives, which subsequently participates in the tricarboxylic acid (TCA) cycle, while the other generates GSH for reactive oxygen species (ROS) scavenging [8]. BCAT1, a pivotal enzyme in BCAA metabolism, has emerged as a focal point in cancer research due to its diverse roles across various malignancies [9]. Jared et, al reported elevated BCAT1 expression in non-small cell lung cancer, and loss of BCAT1 impairs NSCLC tumor formation [10]. In acute myeloid leukemia stem cells, BCAT1 restricts α-ketoglutarate levels and induces DNA hypermethylation, thereby maintaining cancer stem cell function [11]. BCAT1 is also associated with cisplatin sensitivity in hepatocellular carcinoma via mTOR-mediated autophagy [12]. Notably, BCAT1 promotes cell proliferation in aggressive gliomas, and BCAT1 inhibition in combination with α-ketoglutarate triggers metabolic synthetic lethality in glioma cells [13, 14]. The intricate regulatory mechanisms governing BCAT1 expression include transcriptional factors such as c-Myc and HIF-1α [15, 16], and post-transcriptional regulation involving miR-124-3p [17]. Additionally, intracellular location of BCAT1 is regulated by phosphorylation and palmitoylation [18]. However, limited knowledge exists regarding the posttranslational regulation of BCAT1 expression.

In this study, we discover that ubiquitination at Lys360 facilitates BCAT1 degradation, and low ubiquitination levels contribute to high BCAT1 expression in glioma cells. The E3 ligase CHIP binds to BCAT1 through its coiled-coil domain, and promotes BCAT1 K48-linkage ubiquitination. CHIP-mediated BCAT1 degradation via the proteasomal pathway inhibits glioma cell proliferation and tumor formation both in vitro and in vivo. Additionally, low CHIP expression correlates with high BCAT1 expression and poor prognosis in glioma patients. Critically, we also demonstrate that the CHIP/BCAT1 axis enhances temozolomide sensitivity in glioma cells by reducing GSH synthesis and increasing oxidative stress. Collectively, our study reveals a previously unknown regulatory mechanism of BCAT1 post-translational modification in glioma and offers a potential therapeutic target to enhance temozolomide sensitivity.

## Materials and methods

### Cell culture

Human malignant glioblastoma multiforme U251 and U87 cells were cultured in high-glucose Dulbecco’s Modified Eagle Medium supplemented with 10% fetal bovine serum. Human microglial HMC3 cells were cultured in low-glucose Dulbecco’s Modified Eagle Medium supplemented with 10% fetal bovine serum. All cells were maintained in a humidified atmosphere at 37 °C with 5% CO_2_. These cell lines were purchased from the National Collection of Authenticated Cell Cultures of the Chinese Academy of Sciences (Shanghai, China). All cell lines were authenticated using short-tandem repeat analyses by a third-party biotechnology company (Biowing Applied Biotechnology, Shanghai) and all cells were tested without mycoplasma contamination.

### GST-pull down, co-immunoprecipitation (co-IP) and western blot assay

For the GST-pull down assay, recombinant CHIP protein (#HY-P71340, MedChemExpress) was incubated with GSTSep Glutathione Magbeads (#20562ES03, Yeasen Biotechnology) and GST (#HY-P70270, MedChemExpress) or GST-BCAT1 (#Ag4574, Proteintech) protein at 4 °C for 2 h. Subsequently, the beads were washed thrice with PBS and boiled at 100 °C for 10 min with 1×loading buffer.

For the co-IP assay, cells were lysed in NP-40 Lysis buffer and centrifuged at 12,000 × *g* for 20 min at 4 °C. The co-IP system contained 1 μg antibody and 30 μL protein A/G agarose beads, and was incubated on a Rotator Shaker at 4 °C for 8 h. The beads were then washed thrice with Lysis buffer and boiled at 100 °C for 10 min with 1×loading buffer.

For the western blot assay, proteins were separated using Sodium dodecyl-sulfate polyacrylamide gel electrophoresis and transferred onto a polyvinylidene difluoride membrane. The membranes were blocked with 5% fat-free milk and incubated overnight at 4 °C with the following primary antibodies: CHIP (#2080, Cell Signaling Technology), BCAT1(#13640-1-AP, Proteintech), HA (#51064-2-AP, Proteintech), Flag (#66008-4-lg, Proteintech), His (#66005-1-Ig, Proteintech), ubiquitin (#10201-2-AP, Proteintech), cleaved Caspase 3 (#9664 S, Cell Signaling Technology), cleaved Caspase 9 (#9507 S, Cell Signaling Technology) and GAPDH (#10494-1-AP, Proteintech). After washed thrice with Tris-buffered saline containing 0.1% Tween-20, the membranes were incubated with HRP-conjugated secondary antibodies. Protein band were visualized using a chemiluminescence detection kit and acquired with a BioRad digital gel image analysis system (ChemiDoc MP).

### Immunofluorescence assay

To detect the subcellular location of BCAT1 and CHIP, cells were fixed in 4% paraformaldehyde for 20 min at room temperature and permeabilized in phosphate-buffered saline (PBS) containing 0.5% Trinton-X. Subsequently, cells were blocked with 5% goat serum and incubated with the primary antibody overnight at 4 °C. On the following day, cells were washed thrice with PBS containing 0.5% Tween-20 and incubated with fluorescent secondary antibodies (#RGAR004, #RGAM002, Proteintech). Nuclear staining was performed using 4’,6-diamidino-2-phenylindole (DAPI). The images were acquired with an inverted fluorescence microscope (Eclipse Ti2-U, Nikon).

To measure ROS, cells were incubated with 5 μM Dihydroethidium (DHE; #HY-D0079, MedChemExpress) for 30 min at 37 °C in the dark, and washed twice with PBS. The images were acquired using an inverted fluorescence microscope (Eclipse Ti2-U, Nikon), and fluorescence density was quantified using flow cytometry (FACS Canto® II, BD).

### RNA extraction and quantitative real-time PCR

Total RNA was extracted using TRIzol reagent and transcribed into cDNA using the M5 Sprint qPCR RT kit with gDNA remover (MF949-01, Mei5bio). Quantitative real-time PCR was performed using M5 Hiper Realtime Super Mix with High Rox (#MF013-505, Mei5bio). The primer sequences used are listed in Table S1.

### Cell proliferation assay

For the Crystal Violet assay, cells were seeded in a 24-well plate and cultured for the indicated times. Subsequently, cells were fixed in 4% paraformaldehyde and stained with 0.1% crystal violet. The crystal violet stain was then dissolved in 10% acetic acid, and absorbance at 595 nm was measured.

For the colony formation assay, cells were seeded in a 12-well plate and transfected with the indicated plasmids or siRNAs. The cells were then cultured for 10–15 days, fixed in 4% paraformaldehyde and stained with 0.1% crystal violet. The wells were photographed and colony numbers in each well was quantified.

For the EdU staining assay, cells were seed on glass coverslips in a 12-well plate. EdU staining was performed using the EdU Imaging kit (#K1075, APExBio) according to manufacturer’s instructions. Images were acquired with an inverted fluorescence microscope (Eclipse Ti2-U, Nikon).

### Stable cell line construction

Full length CHIP and K360R mutated BCAT1 sequences were cloned into pCDH-CMV-puro or pLV-EF1α-Bsd plasmids. The recombinant plasmids were co-transfected with psPAX2 and pMD2.G plasmids into HEK-293 cells for lentivirus packaging. U87 cells were then transduced with lentiviral stocks and screened with puromycin or Blasticidin S, respectively. The primer sequences are listed in Table S1.

### In vivo xenograft assay

Four-week-old male NOD/SCID mice were purchased from SPF (Beijing) Biotechnology Co., Ltd. Mice were randomly divided into different groups, subcutaneously injected with a total number of 3 × 10^6^ cells and housed in a Specific Pathogen Free animal facility. Temozolomide was administered intraperitoneally every other day. After the indicated times, the mice were sacrificed and tumor tissues were excised. The investigator was blinded to the group allocation of the mice during the experiment. No animals were excluded from the analysis. This study was approved by the Ethics Committee of Jiangxi Provincial People’s Hospital (protocol code KT2022-001).

### Hematoxylin-eosin (H&E) staining and immunohistochemistry (IHC) staining

H&E staining and IHC staining of xenografted tumors and tumor microarrays were conducted by Wuhan ServiceBio Technology Co., Ltd.

### Metabolomic analysis

Metabolite content in cells was determined using quadrupole time-of-flight (Q-TOF) liquid chromatography (LC)/mass spectrometry (MS) conducted by MagiGene Biotechnology.

### Apoptosis assay

Cell apoptosis were assessed using Annexin V-FITC/PI Apoptosis Kit (#E-CK-A211, Elabscience). After the indicated treatments, cells were digested with EDTA-free trypsin and resuspended in 100 μL of 1×Annexin V Binding Buffer. Annexin V-FITC reagent (2.5 µL) and propidium Iodide (PI) reagent (2.5 µL) were then added to each sample, followed by incubation at room temperature for 20 min in the dark. Each sample was then mixed with 400 μL of 1×Annexin V Binding Buffer and analyzed using flow cytometry (FACS Canto® II, BD).

### GSH content measurement

Intracellular GSH content was quantified using Reduced Glutathione Content Assay Kit (#BC1175, Solarbio) according the manufacturer’s instructions.

### Statistical analysis

Data are shown as mean ± SD. Statistical differences between different groups were assessed by Student’s *t* test or one-way analysis of variance (ANOVA) followed by Fisher’s LSD test where appropriate. A *p* value of <0.05 was considered statistically significant.

## Results

### Ubiquitination at Lys360 facilitates BCAT1 degradation via the proteasomal pathway

**Fig. 1:**
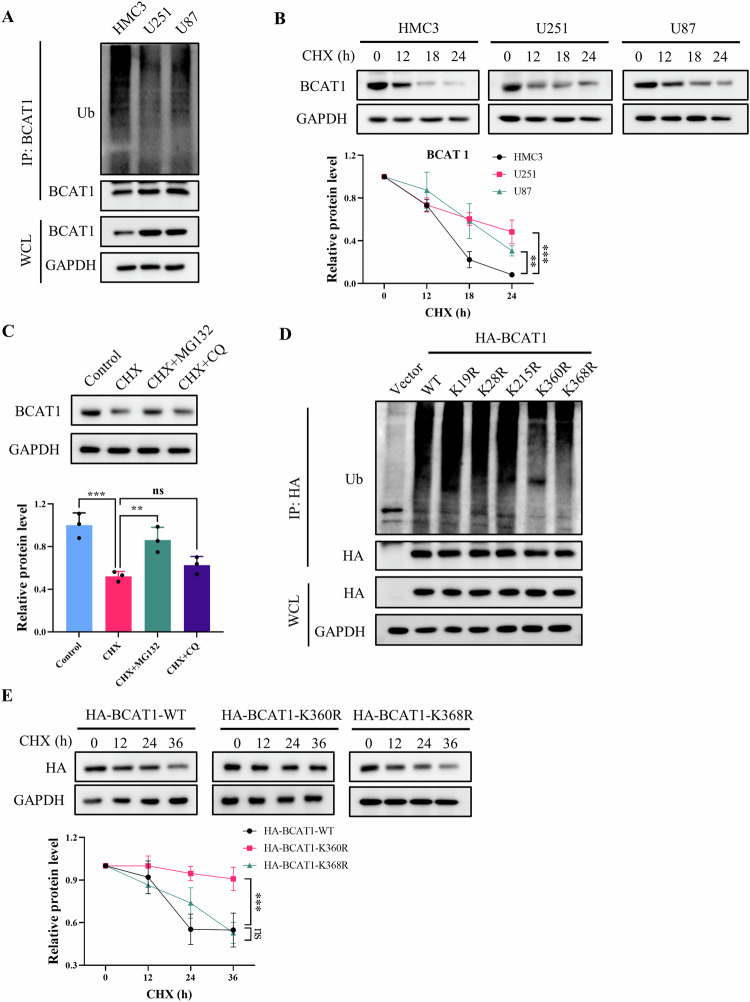
Ubiquitylation at Lys360 promotes BCAT1 degradation through proteasomal pathway. **A** BCAT1 ubiquitination levels in HMC3, U251 and U87 cells were detected by immunoprecipitation and western blot. **B** HMC3, U251 and U87 cells were treated with 50 µg/mL cycloheximide, and BCAT1 expression was detected by western blot. **C** U251 cells were treated with 50 µg/mL cycloheximide with or without 10 µM MG132 or 10 µM chloroquine for 18 h, and BCAT1 expression was detected by western blot. **D** U251 cells were transfected with HA-tagged wild-type BCAT1 or BCAT1 mutant plasmid, and BCAT1 ubiquitination was detected by immunoprecipitation and western blot. **E** U251 cells were transfected with HA-tagged wild-type BCAT1 plasmid or BCAT1 mutant plasmid, and treated with 50 µg/mL cycloheximide. HA-BCAT1 expression was detected by western blot. Data were presented as mean ± SD of three independent experiments. ns *p* > 0.05, **p < 0.01, ***p < 0.001. CHX cycloheximide, CQ chloroquine.

To elucidate the regulatory mechanism governing BCAT1 post-translational modification and expression in glioma cells, we conducted a ubiquitin assay to evaluate BCAT1 ubiquitination levels in human malignant glioblastoma multiforme U251 and U87 cells, as well as in human microglial HMC3 cells. The results revealed higher BCAT1 ubiquitylation levels in HMC3 cells compared to U251 and U87 cells (Fig. 1A). A cycloheximide chase assay demonstrated a significantly prolonged half-life of BCAT1 in U251 and U87 cells compared to HMC3 cells (Fig. 1B). Furthermore, BCAT1 turnover was impeded by MG-132, a proteasome inhibitor, but not by chloroquine (CQ), a lysosome inhibitor (Fig. 1C), indicating the involvement of the ubiquitin-proteasome pathway in BCAT1 degradation. Previous studies identified several ubiquitylated lysine residues on BCAT1, specifically Lys19, Lys28, Lys215, Lys360 and Lys368. In this study, plasmids containing K19R, K28R, K215R, K360R, K368R or wild-type BCAT1 were constructed and transfected into U251 cells, and BCAT1 ubiquitylation levels were assessed. The results showed that K360R and K368R mutations reduced BCAT1 ubiquitylation levels in U251 cells (Fig. 1D). Moreover, the half-life of BCAT1^K360R^ was significantly prolonged compared to wild-type BCAT1 and BCAT1^K368R^ mutant (Fig. 1E), suggesting that K360 is the major ubiquitination site regulating BCAT1 stability. These results indicate that the heightened expression of BCAT1 in glioma cells is regulated by ubiquitination and proteasomal degradation.

### CHIP interacts with BCAT1 to facilitate its ubiquitination and degradation

**Fig. 2:**
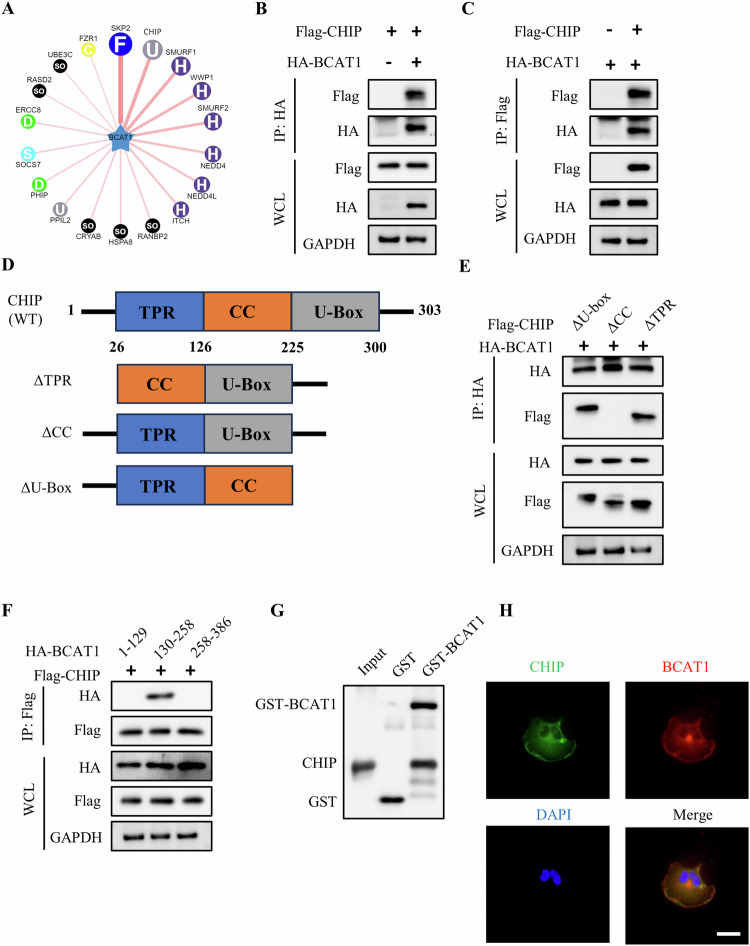
CHIP binds to BCAT1 through coiled-coil domain. **A** Predicted ubiquitin ligase for BCAT1 on http://ubibrowser.bio-it.cn/ubibrowser/. **B**, **C** U251 cells were transfected with HA-BCAT1 with or without Flag-CHIP plasmid. The interaction between BCAT1 and CHIP was detected by immunoprecipitation and western blot. **D** Schematic diagram of wile-type CHIP and its truncated mutants. **E** U251 cells were transfected with HA-BCAT1 plasmid with or without Flag-tagged wild-type CHIP or CHIP mutant plasmid, and immunoprecipitation and western blot was performed. **F** U251 cells were transfected with Flag-tagged CHIP plasmid and HA-tagged wild-type BCAT1 or BCAT1 mutant plasmid, and immunoprecipitation and western blot was performed. **G** Subcellular location of BCAT1 and CHIP was detected by immunofluorescence. Scale bar = 50 μm. **H** In vitro binding assay of GST-BCAT1 and CHIP proteins.

To investigate the underlying mechanism of BCAT1 ubiquitylation in glioma cells, potential E3 ligase for BCAT1 was screened using UbiBroswer, an integrated online database for predicting human E3–substrate interactions (Fig. 2A). co-IP and western blot analyses confirmed the interaction between CHIP and BCAT1 in U251 cells (Fig. 2B, C), while no interaction was detected between BCAT1 and SKP2 or Smurf2 (Fig. S1A, B). To delineate the interaction domain of CHIP with BCAT1, three CHIP truncation plasmids were constructed (Fig. 2D). co-IP and western blot results demonstrated that CHIP mutants lacking U-box and TPR domains bound to BCAT1, whereas the mutant lacking the CC domain did not (Fig. 2E), indicating that CHIP interacts with BCAT1 through the CC domain. Additionally, BCAT1 truncation plasmids were constructed and co-transfected with Flag-CHIP plasmid. co-IP and western blot results indicated that CHIP binds to 130-258 amino acid residues of BCAT1 (Fig. 2F). A GST pull-down assay demonstrated in vitro interaction between GST-BCAT1 and CHIP (Fig. 2G). An immunofluorescence assay validated the cytoplasmic co-localization of CHIP and BCAT1 (Fig. 2H). These findings collectively establish that CHIP directly binds to BCAT1.

**Fig. 3:**
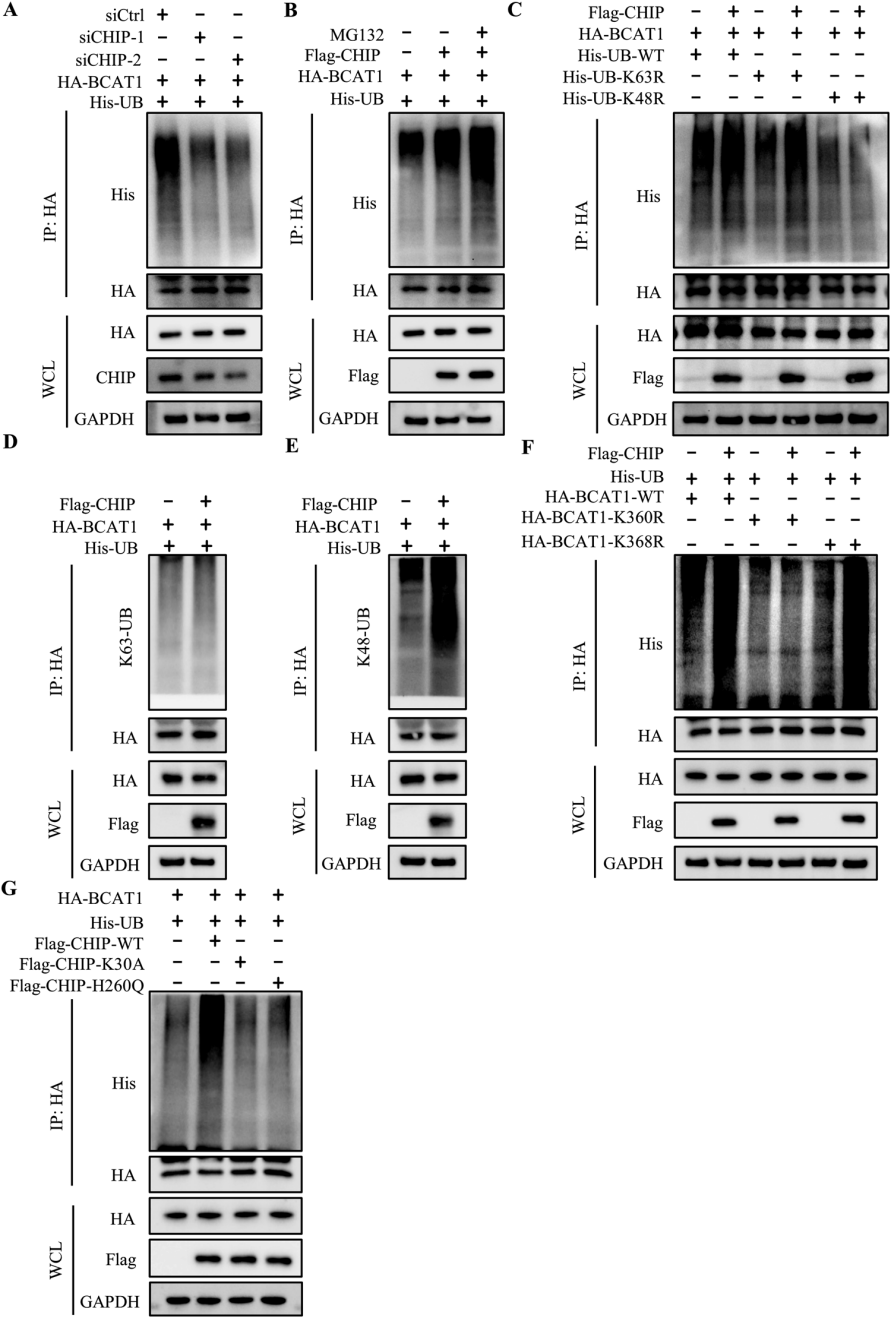
CHIP induces K48-linkage BCAT1 ubiquitylation at Lys360. **A** U251 cells were co-transfected with HA-BCAT1 and His-ubiquitin plasmid with or without CHIP siRNA. BCAT1 ubiquitination was detected by immunoprecipitation and western blot. **B** U251 cells were co-transfected with HA-BCAT1 and His-ubiquitin plasmid with or without Flag-CHIP plasmid. Cells were then treated with 10 µM MG132 for 12 h, and BCAT1 ubiquitination was detected by immunoprecipitation and western blot. **C** U251 cells were co-transfected with HA-BCAT1 and His-ubiquitin plasmid with or without Flag-CHIP plasmid, and BCAT1 ubiquitination was detected by immunoprecipitation and western blot. **D**, **E** U251 cells were co-transfected with HA-BCAT1 and His-ubiquitin plasmid with or without Flag-CHIP plasmid. BCAT1 ubiquitin chain linkage was detected by immunoprecipitation and western blot. **F** U251 cells were co-transfected with HA-tagged wild-type BCAT1 plasmid or BCAT1 mutant plasmid and His-ubiquitin, Flag-CHIP plasmid. BCAT1 ubiquitination was detected by immunoprecipitation and western blot. **G** U251 cells were transfected with HA-BCAT1, His-ubiquitin plasmid and Flag-tagged wild-type CHIP plasmid or CHIP mutant plasmid. BCAT1 ubiquitination was detected by immunoprecipitation and western blot.

Given CHIP’s role as an E3 ligase, we explored whether CHIP regulates BCAT1 ubiquitylation. CHIP knockdown by siRNA decreased BCAT1 ubiquitylation in U251 cells (Fig. 3A). Conversely, CHIP overexpression enhanced BCAT1 ubiquitylation, and MG-132 treatment further increased BCAT1 ubiquitylation, affirming the involvement of proteasomal pathway in BCAT1 degradation (Fig. 3B). We then examined the type of ubiquitin chain on BCAT1 catalyzed by CHIP, and the results showed that CHIP overexpression increased K48-linkage ubiquitylation of BCAT1 rather than K63-linkage ubiquitylation (Fig. 3C–E). Moreover, CHIP promoted ubiquitylation of BCAT1^K368R^ mutant without affecting the ubiquitylation of BCAT1^K360R^ mutant (Fig. 3F). K30 and H260 are two key enzymatic sites of CHIP. Overexpression of CHIP K30A or H260Q mutant lost the ability to promote BCAT1 ubiquitylation (Fig. 3G), suggesting that CHIP regulates BCAT1 ubiquitylation in an E3 ligase activity-dependent manner. In summary, these results demonstrate that CHIP facilitates K48-linkage ubiquitylation of BCAT1 at Lys360.

**Fig. 4:**
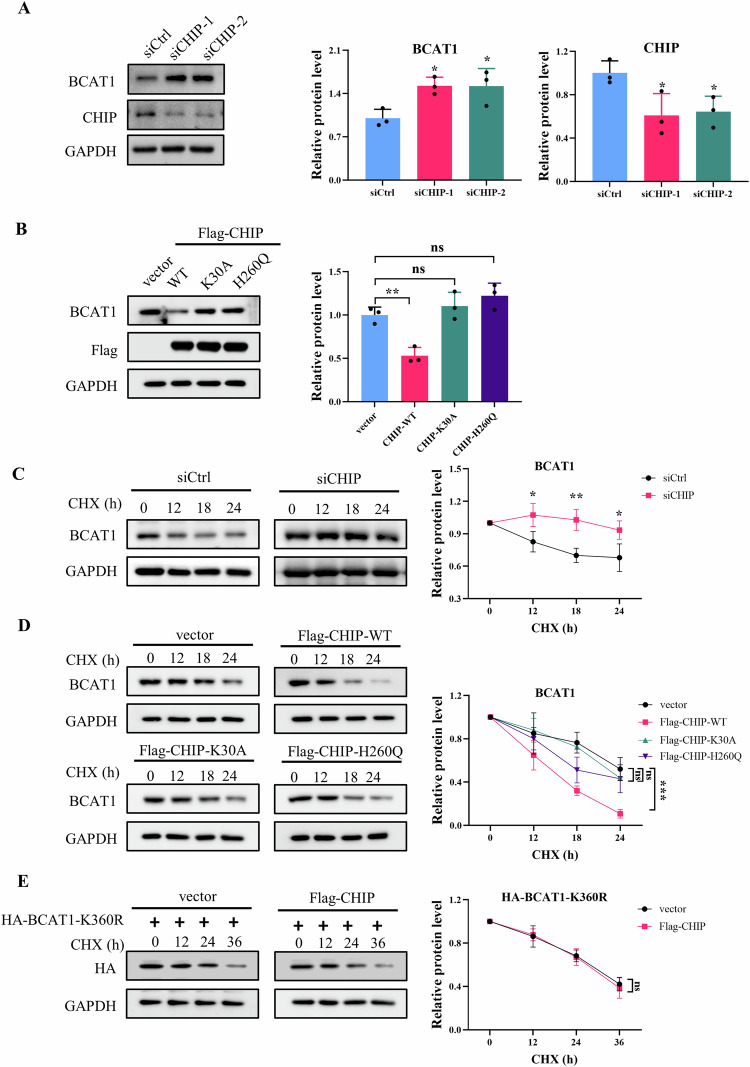
CHIP promotes BCAT1 degradation. **A** U251 cells were transfected with scramble siRNA or CHIP siRNA. BCAT1 and CHIP expression were detected by western blot. **B** U251 cells were transfected with Flag-tagged wild-type CHIP plasmid or CHIP mutant plasmid. BCAT1 and CHIP expression were detected by western blot. **C** U251 cells were transfected with scramble siRNA or CHIP siRNA, and treated with 50 µg/mL cycloheximide. BCAT1 expression were detected by western blot. **D** U251 cells were transfected with Flag-tagged wild-type CHIP plasmid or CHIP mutant plasmid, and treated with 50 µg/mL cycloheximide. BCAT1 expression were detected by western blot. **E** U251 cells were transfected with HA-BCAT-K360R plasmid with or without Flag-CHIP plasmid, and treated with 50 µg/mL cycloheximide. BCAT1 expression were detected by western blot. Data were presented as mean ± SD of three independent experiments. ns *p* > 0.05, *p < 0.05, **p < 0.01, ***p < 0.001.

Next, we examined whether CHIP-mediated BCAT1 ubiquitylation facilitates BCAT1 degradation. CHIP knockdown significantly upregulated BCAT1 expression (Fig. 4A). Conversely, overexpression of wild-type CHIP, but not the CHIP K30A or H260Q mutant, downregulated BCAT1 expression (Fig. 4B). Results of RT-qPCR indicated that neither CHIP overexpression nor knockdown affected BCAT1 transcription (Fig. S2A–D). Furthermore, the cycloheximide chase assay revealed that CHIP knockdown extended the half-life of BCAT1 (Fig. 4C). Overexpression of wild-type CHIP, but not CHIP mutants, promoted BCAT1 turnover (Fig. 4D). Additionally, CHIP overexpression had no impact on the half-life of BCAT1^K360R^ mutant (Fig. 4E). These findings collectively signify that CHIP promotes BCAT1 degradation through ubiquitylation at Lys360.

### CHIP-mediated BCAT1 degradation suppresses cell proliferation and tumor growth

**Fig. 5:**
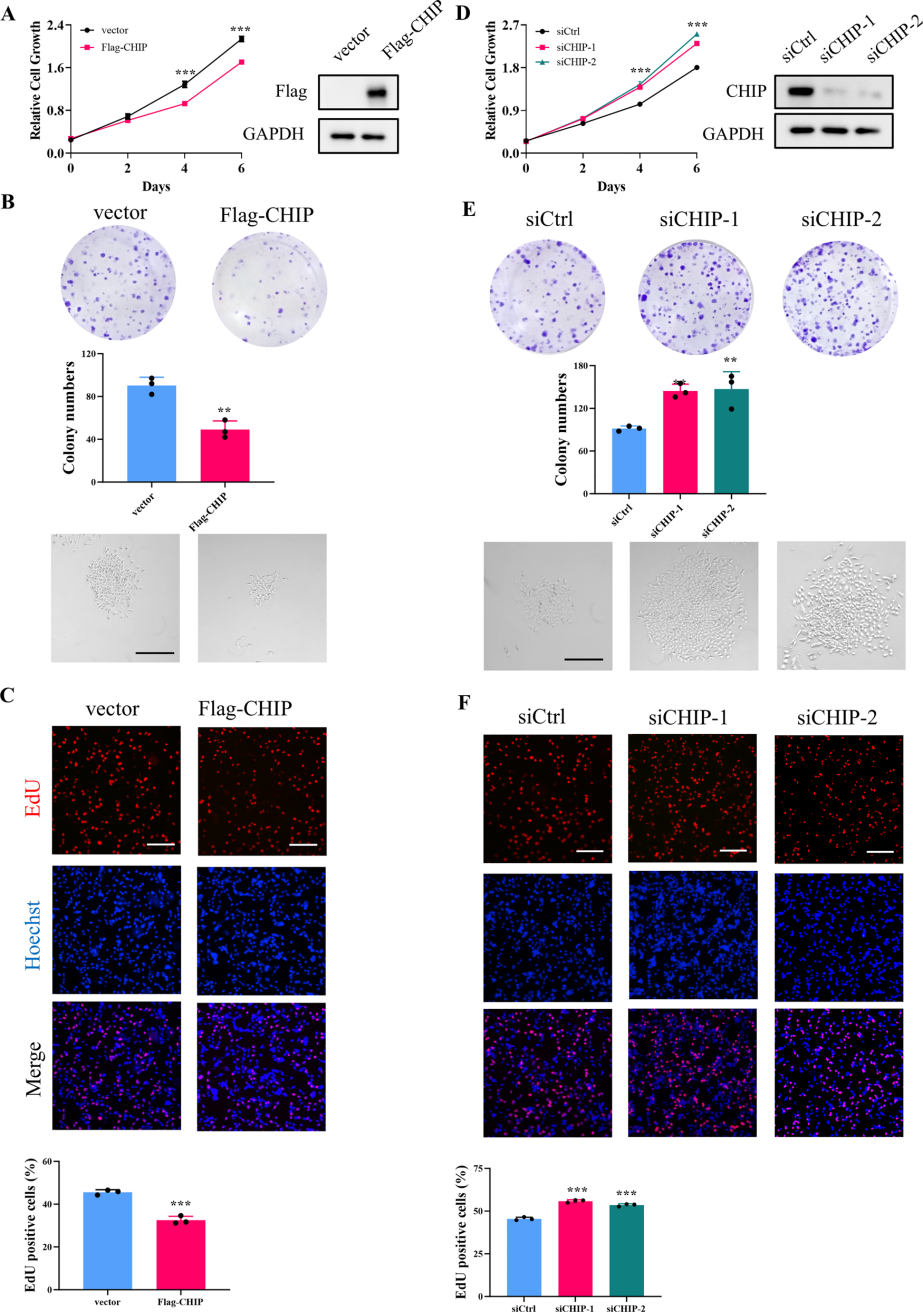
CHIP inhibits glioblastoma cell proliferation. **A**–**C** U251 cells were transfected with vector or Flag-CHIP plasmid. Cell proliferation and colony formation were detected by crystal violet staining and EdU staining. **D**–**F** U251 cells were transfected with scramble siRNA or CHIP siRNA. Cell proliferation and colony formation were detected by crystal violet staining and EdU staining. Scale bar = 200 μm. Data were presented as mean ± SD of three independent experiments. ***p < 0.001.

Considering CHIP’s dualistic roles in various cancer types [19, 20], we investigated its biological function in glioma cells. Crystal violet and EdU staining demonstrated that CHIP overexpression inhibited the proliferation of U251 and U87 cells, and impaired colony formation ability in U251 cells (Fig. 5A–C; Fig. S3A, B). Conversely, CHIP knockdown promoted proliferation and colony formation in glioma cells (Fig. 5D–F; Fig. S3C, D), suggesting CHIP acts as a tumor suppressor in glioma.

**Fig. 6:**
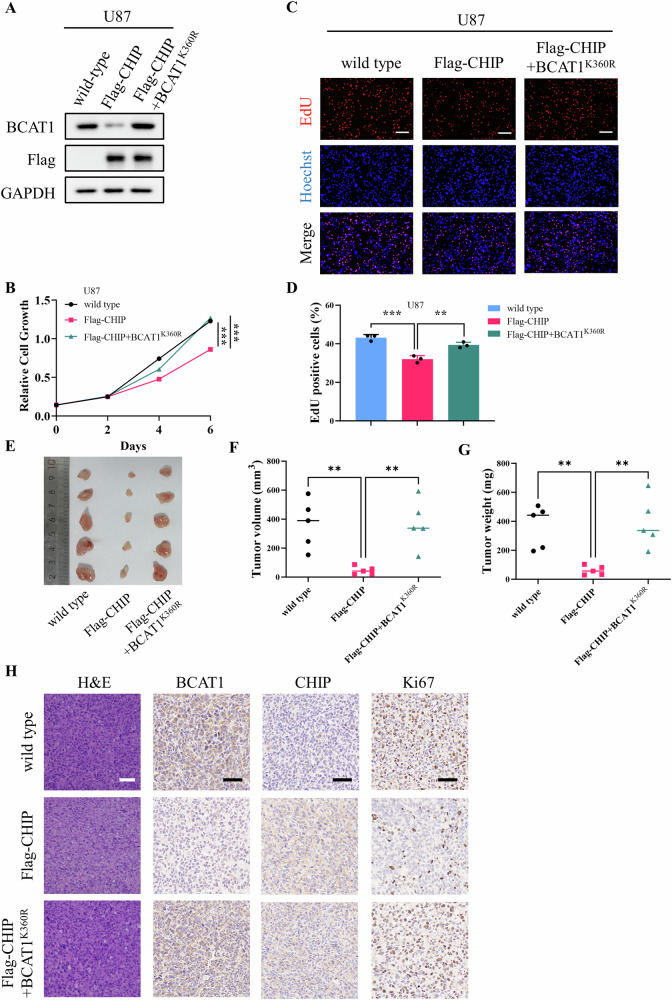
CHIP-mediated BCAT1 degradation inhibits tumor growth in xenograft model. **A** U87 cells stably expressing CHIP and BCAT1^K360R^ were constructed. **B**–**D** Cell proliferation was detected by crystal violet staining and EdU staining. **E**–**G** Male NOD/SCID mice were subcutaneously injected with wild-type U87 cells, U87-CHIP cells or U87-CHIP/BCAT1^K360R^ cells. Fourteen days later, mice were sacrificed and tumors were dissected and photographed (**E**). Volume (**F**) and weight (**G**) of xenograft tumors were measured. *n* = 5. **H** H&E staining and immunohistochemistry analysis of the sections of tumor. Scale bar = 50 μm. Data were presented as mean ± SD. ** *p* < 0.01, ***p < 0.001.

To further validate the functional impact of CHIP in glioma cells, stable U87 cell lines overexpressing Flag-CHIP and BCAT1^K360R^ were generated (Fig. 6A). Functional experiments revealed that the proliferative capacity of U87-CHIP cells was suppressed compared to wild-type U87 cells, and constitutive expression of BCAT1^K360R^ restored the proliferative ability of U87-CHIP cells (Fig. 6B-D). Subsequently, xenograft assays were performed to assess the tumorigenesis of wild-type U87 cells and U87-CHIP, U87-CHIP/BCAT1^K360R^ cells. As depicted in Fig. 6E–G, NOD SCID mice xenografted with U87-CHIP cells exhibited reduced tumor volume and weight compared to those xenografted with wild-type U87 cells. However, mice xenografted with U87-CHIP/BCAT1^K360R^ cells displayed a resumption of tumor volume and weight, confirming that CHIP inhibited tumor growth through BCAT1 degradation. To further substantiate these findings, H&E and IHC staining was performed (Fig. 6H). The expression of CHIP was markedly higher in U87-CHIP and U87-CHIP/BCAT1^K360R^ groups. Low BCAT1 expression was observed in U87-CHIP group, while high BCAT1 expression was observed in U87-CHIP/BCAT1^K360R^ group. Additionally, the expression of Ki67, a cell proliferation marker, was remarkedly lower in U87-CHIP group and higher in U87-CHIP/BCAT1^K360R^ group. Collectively, these results underscore that CHIP-mediated BCAT1 degradation effectively inhibits tumor growth in vivo.

### CHIP-mediated BCAT1 degradation induces metabolic reprogramming

**Fig. 7:**
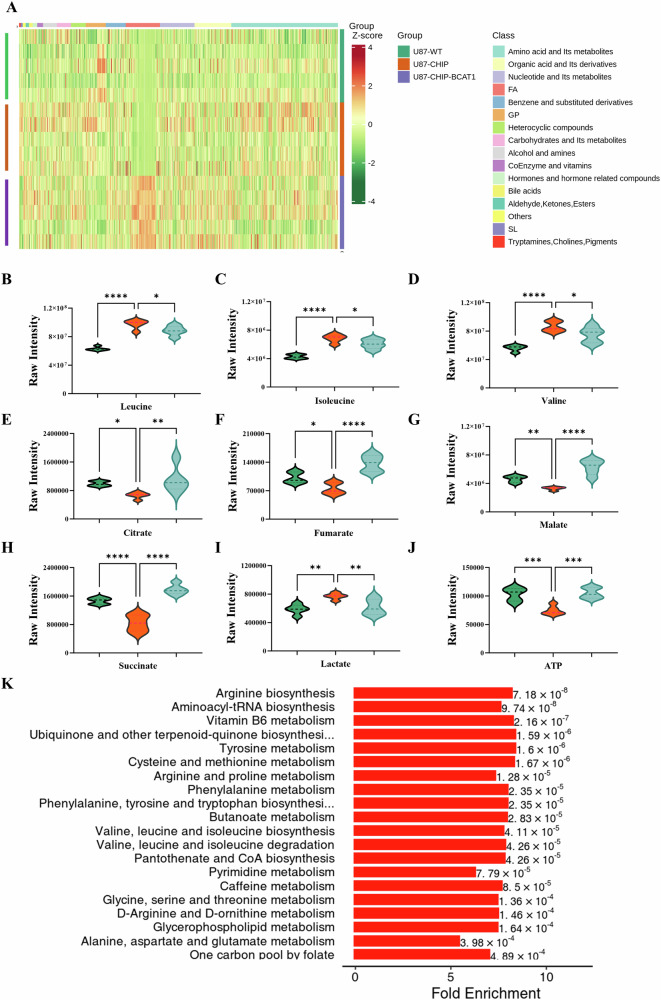
CHIP-mediated BCAT1 degradation induces metabolic reprogramming. Extracts from wild-type U87 cells, U87-CHIP and U87-CHIP/BCAT1^K360R^ cells were analyzed using an LC-ESI-MS/MS system. **A** Heatmap showing a visualization of clustering analyses of metabolites identified from cell lines. **B**–**J** Relative levels of leucine, isoleucine, valine, citrate, fumarate, malate, succinate, lactate and ATP in different cell lines. **K** KEGG pathway analysis of the metabolomic data. Data were presented as mean ± SD, *n* = 5. * *p* < 0.05, ** *p* < 0.01, ***p < 0.001.

We further employed metabolomics analysis to elucidate the metabolic alterations associated with varying tumorigenic potential. Concentrations of various metabolites in wild-type U87 cells, U87-CHIP and U87-CHIP/BCAT1^K360R^ cells were assessed, revealing 467 differentially abundant metabolites (Figs. 7A and S4). Specifically, intracellular contents of leucine, isoleucine and valine were elevated in U87-CHIP cells compared to wild-type U87 cells, and diminished in U87-CHIP/BCAT1^K360R^ cells, indicating suppressed BCAA metabolism in U87-CHIP cells (Fig. 7B–D). Furthermore, levels of citrate, fumarate, malate and succinate were reduced in U87-CHIP cells, suggesting inhibition of TCA cycle (Fig. 7E–H). Notably, lactate levels were increased in U87-CHIP cells, suggesting a potential shift to glycolysis (Fig. 7I). Additionally, ATP levels were lower in U87-CHIP cells compared to wild-type U87 and U87-CHIP/BCAT1^K360R^ cells, indicative of impaired energy production (Fig. 7J). KEGG pathway analysis revealed significant alterations in most amino acid metabolism pathways (Fig. 7K). These findings collectively demonstrate that CHIP-mediated BCAT1 degradation induces metabolic reprogramming.

### Low CHIP expression correlates with high BCAT1 expression and poor prognosis in glioma patients

**Fig. 8:**
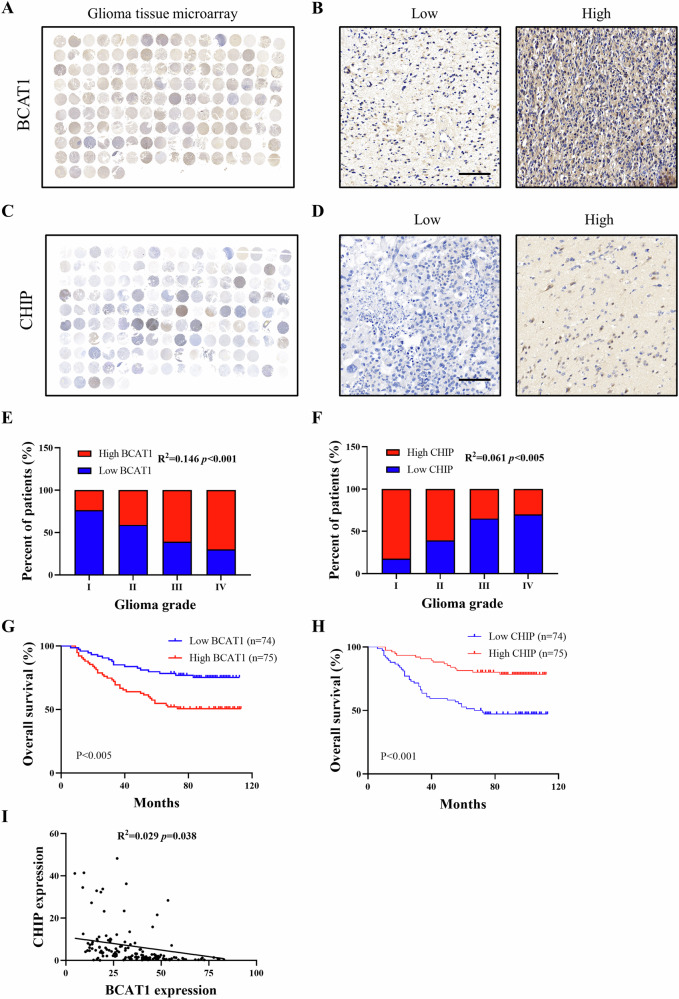
Low CHIP expression correlates with high BCAT1 expression and poor prognosis of glioblastoma patients. **A** Immunohistochemistry staining of human glioma tissue microarray with anti-BCAT1 antibody. **B** Representative images of immunohistochemistry staining of BCAT1. **C** Immunohistochemistry staining of human glioma tissue microarray with anti-CHIP antibody. **D** Representative images of immunohistochemistry staining of CHIP. **E**, **F** Concomitantly high BCAT1 expression (**E**) and low CHIP expression (**F**) positively correlates with advanced glioma grades. **G**, **H** Kaplan-Meier survival curve of 149 patients in the glioma tissue microarray based on BCAT1 and CHIP expression. **I** Correlation between BCAT1 and CHIP expression in glioma tissue microarrays. Scale bar = 100 μm.

To delve further into the clinical relevance of CHIP and BCAT1 expression in glioma patients, their expression was assessed in glioma tissue microarrays (Fig. 8A–D). The analysis revealed concomitantly high BCAT1 expression and low CHIP expression in patients with advanced glioma grades (Fig. 8E, F). Survival analysis indicated that glioma patients exhibiting high BCAT1 expression and low CHIP expression had shorter overall survival compared to those with low BCAT1 expression and high CHIP expression (Fig. 8G, H). Moreover, BCAT1 and CHIP expression displayed a negative correlation in glioma tissue microarray samples (Fig. 8I). In sum, these data suggest that aberrant expression of BCAT1 and CHIP contributes to glioma progression and can serve as prognostic markers for glioma patients.

### CHIP-mediated BCAT1 degradation enhances the sensitivity of glioma to temozolomide

**Fig. 9:**
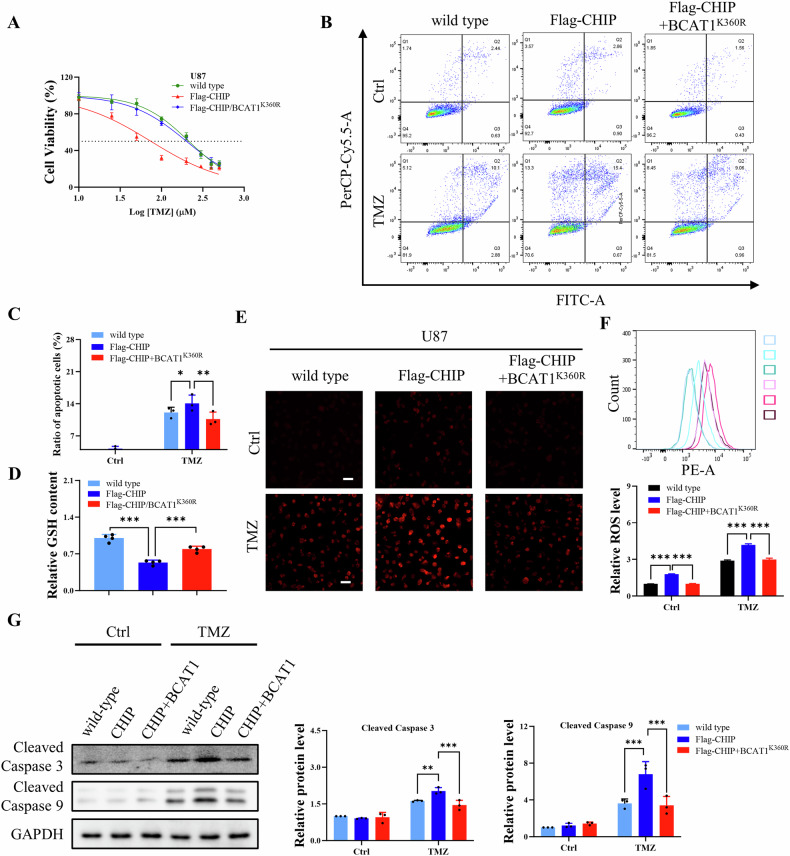
CHIP-mediated BCAT1 degradation enhances temozolomide sensitivity in glioma cells. **A** IC50 values of temozolomide for the wild-type U87, U87-CHIP and U87-CHIP/BCAT1^K360R^ cells. **B** wild-type U87, U87-CHIP and U87-CHIP/BCAT1^K360R^ cells were treated with 100 μM temozolomide for 48 h. The ratio of apoptotic cells was detected by Annexin/PI staining and flow cytometry. **C** Quantification of the ratio of apoptotic cells. **D** Relative contents of GSH in wild-type U87, U87-CHIP and U87-CHIP/BCAT1^K360R^ cells. **E**–**G** Wild-type U87, U87-CHIP and U87-CHIP/BCAT1^K360R^ cells were treated with 100 μM temozolomide for 48 h. Intracellular ROS level was detected by DHE staining. Representative immunofluorescence images were shown (**E**), and fluorescence density was quantified by flow cytometry (**F**). Scale bar = 100 μm. The expression of Cleaved Capase-3 and Cleaved Caspase-9 was detected by western blot (**G**). Data were presented as mean ± SD of three independent experiments. ***p < 0.001. TMZ temozolomide.

Temozolomide, a first-line chemotherapy drug for glioma patients, faces longstanding challenges of resistance in clinical settings. To investigate whether CHIP/BCAT1 axis correlates with the sensitivity of glioma cells to temozolomide, we examined the IC50 values of temozolomide using CCK-8 assay. The results revealed IC50 values of 214.8, 74.6 and 200.9 μM in wild-type U87, U87-CHIP and U87-CHIP/BCAT1^K360R^ cells, respectively (Fig. 9A). Similar trends were observed in U251 cells (Fig. S5A). Moreover, BCAT1 knockdown decreased the IC50 value of temozolomide in U251 cells (Fig. S5B, C), while BCAT1 overexpression conferred resistance to temozolomide in U251 cells (Fig. S5D). Additionally, U87-CHIP cells treated with temozolomide exhibited a higher ratio of apoptotic cells in comparison to wild-type U87 cells and U87-CHIP/BCAT1^K360R^ cells (Fig. 9B, C), indicating that CHIP-mediated BCAT1 degradation promoted temozolomide sensitivity in glioma cells.

Prior research indicates that BCAA metabolism contributes to glutathione (GSH) synthesis, a crucial element in maintaining cellular redox homeostasis [8]. To investigate whether the CHIP/BCAT1 axis regulates temozolomide sensitivity through GSH synthesis and oxidative stress, we assessed GSH contents in wild-type U87 cells and U87 stable cell lines. The results showed a significantly lower GSH level in U87-CHIP cells compared to wild-type U87 cells and U87-CHIP/BCAT1^K360R^ cells (Fig. 9D). Additionally, BCAT1 knockdown decreased GSH levels in U251 cells (Fig. S5E). Furthermore, temozolomide treatment significantly increased intracellular ROS levels in U87-CHIP cells compared to wild-type U87 cells and U87-CHIP/BCAT1^K360R^ cells (Fig. 9E–F), and induced elevated ROS production in BCAT1 knockdown U251 cells (Fig. S5F). Treatment with NAC, a ROS scavenger, alleviated temozolomide-induced apoptosis in U87-CHIP cells (Fig. S5G). Western blot results showed that temozolomide treatment also upregulated the expression of cleaved Caspase 3 and Caspase 9 (Fig. 9G), indicating the activation of the intrinsic apoptosis pathway. These results suggest that the CHIP/BCAT1 axis regulates temozolomide sensitivity through mitigating oxidative stress via GSH synthesis.

**Fig. 10:**
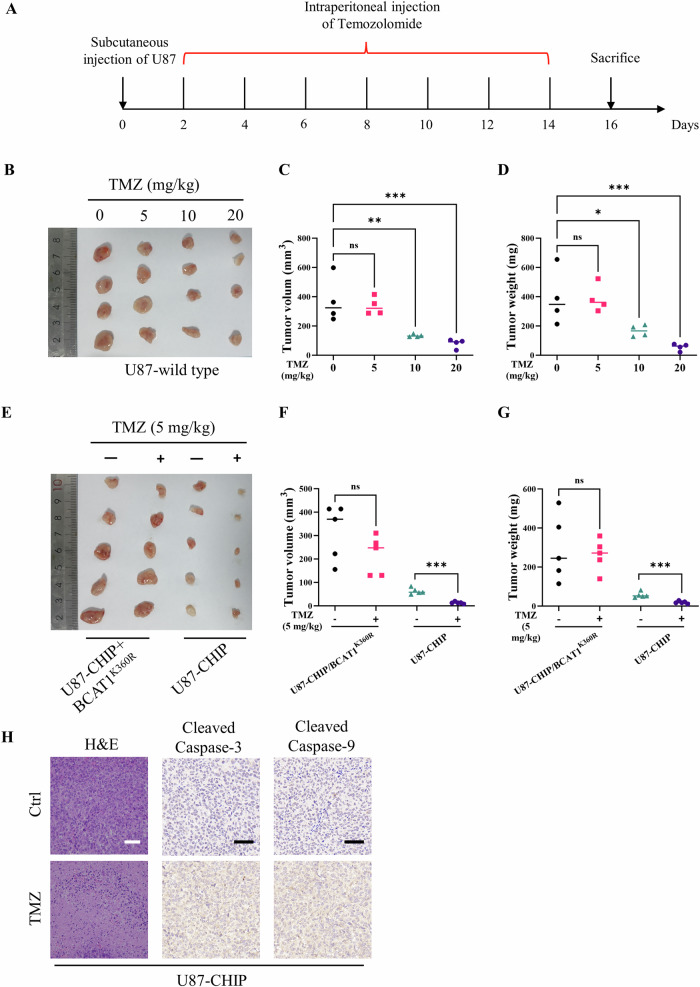
CHIP-mediated BCAT1 degradation enhances temozolomide sensitivity in xenograft model. **A** Schematic diagram of xenograft model and temozolomide administration. (B-D) NOD SCID mice were subcutaneously injected with wild-type U87 cells and intraperitoneally injected with temozolomide. *n* = 4. Image of excised tumors was shown (**B**). Tumor volume (**C**) and weight (**D**) was measured. **E**–**G** NOD SCID mice were subcutaneously injected with U87-CHIP or U87-CHIP/BCAT1^K360R^ cells and intraperitoneally injected with temozolomide. *n* = 5. Image of excised tumors was shown (**E**). Tumor volume (**F**) and weight (**G**) was measured. **H** H&E staining and immunohistochemistry staining of cleaved Caspase-3 and cleaved Caspase-9 in tumor sections were shown. Scale bar = 50 μm. Data were presented as mean ± SD. ns *p* > 0.05, **p < 0.05, **p < 0.01, ***p < 0.001.

Subsequently, xenograft assay was conducted to assess the translational significance of the CHIP/BCAT1 axis in glioma treatment (Fig. 10A). Male NOD SCID mice were subcutaneously injected with wild-type U87 cells and intraperitoneally administered different doses of temozolomide. The results showed that administration of 5 mg/kg temozolomide had no significant effect on xenografted tumor volume and weight, while injection of 10 mg/kg or 20 mg/kg temozolomide significantly inhibited tumor growth (Fig. 10B–D). To validate the role of CHIP/BCAT1 axis in temozolomide sensitivity, NOD SCID mice were xenografted with U87-CHIP or U87-CHIP/BCAT1^K360R^ cells and administrated with 5 mg/kg temozolomide. The results showed that injection of 5 mg/kg temozolomide significantly suppressed xenograft growth of U87-CHIP cells, while the xenograft growth of U87-CHIP/BCAT1^K360R^ cells was unaffected (Fig. 10E–G). H&E and IHC staining demonstrated necrosis and upregulated expression of cleaved Caspase 3 and Caspase 9 in the xenograft tumor of U87-CHIP cells following temozolomide administration (Fig. 10H). Collectively, these results indicate that CHIP-mediated BCAT1 degradation may effectively enhance temozolomide sensitivity in vivo.

## Discussion

Elevated amino acid metabolism plays critical roles in cancer development and progression, contributing significantly to proliferation, metastasis and survival [21]. BCAAs, namely leucine, isoleucine and valine, are essential amino acids for protein synthesis, energy production and various metabolic pathways. Cancer cells often exhibit increased uptake and metabolism of BCAAs to sustain their rapid growth and proliferation, involving increased expression and activity of enzymes like BCAT1 [22], which catalyzes the reversible transamination reaction between BCAAs and α-ketoglutarate, yielding their respective α-keto acids and glutamate [23]. Enhanced BCAT1 expression correlates with aggressive characteristics in various cancers, including glioma, where it associates with unfavorable patient prognosis [14, 24]. Importantly, our previous study demonstrated that targeted inhibition of BCAA metabolism and BCAT1 induces apoptosis in glioma both in vitro and in vivo [25]. Nevertheless, the mechanisms driving this elevation remain elusive. Our investigation unveils that in comparison to microglial cells, glioma cells exhibit lower levels of BCAT1 ubiquitination, leading to an extended half-life. Notably, we identify Lys360 as a pivotal ubiquitination site in BCAT1, promoting its degradation via the proteasomal pathway. Furthermore, the BCAT1^K360R^ mutant displays prolonged stability, linking ubiquitin modification to heightened BCAT1 expression in glioma.

Ubiquitination, a post-translational modification process, involves attaching ubiquitin, a small protein, to lysine residues of target proteins. This modification is catalyzed by a cascade of enzymes including ubiquitin-activating enzymes (E1), ubiquitin-conjugating enzymes (E2), and ubiquitin ligases (E3) [26]. In the past decades, E3 ligases were reported to play crucial roles in maintaining cellular homeostasis by regulating protein turnover through the ubiquitin-proteasome system [27]. Aberrant expression of E3 ligases has been implicated in tumor development and progression by targeting tumor suppressors or oncogenes [28]. For instance, TRIM26 is overexpressed in glioma and suppresses ferroptosis via the degradation of GPX4 [29], while RNF139 inhibits glioma cell growth through PI3K/AKT signaling in a ubiquitination-dependent manner [30]. CHIP, comprising tetratricopeptide repeat (TPR) domain, coiled coil (CC) domain and U-box domains, is involved in protein quality control [31]. Functioning as an E3 ligase through its U-box domain and as a protein chaperone via the TPR domain, CHIP connects the ubiquitin-proteasome system with molecular chaperone machinery [32]. Recognized as a multifaceted regulator in various physiological and pathological processes, CHIP has emerged as a tumor suppressor or oncogene in different cancers by mediating substrate protein degradation [33]. In lung cancer, CHIP stabilizes OTUD3 to suppress metastasis, while in prostate cancer, it enhances sensitivity to enzalutamide and abiraterone by degrading AR/AR-V7 [34, 35]. Notably, in gastric cancer, CHIP facilitates YAP ubiquitination and destabilization, thereby enhances chemotherapy sensitivity [36]. Therefore, identifying novel target is essential for understanding the biological functions of CHIP in glioma. Here, we demonstrated that the CC domain of CHIP interacts with BCAT1. CHIP promotes K48-linked ubiquitination of BCAT1 at Lys360 and its subsequent proteasomal degradation. Importantly, CHIP inhibits the proliferation of glioma cells both in vitro and in vivo, which is rescued by ectopic expression of BCAT1. Moreover, CHIP expression negatively correlates with BCAT1 expression in glioma tissues, with low CHIP expression correlating with the poor prognosis for glioma patients. These results indicate that CHIP targets BCAT1 for ubiquitin and degradation, thereby inhibiting the growth and progression of glioma. Our findings also propose CHIP as a potential biomarker for glioma prognosis.

Temozolomide is an alkylating agent and commonly used as a first-line drugs for glioblastoma [37]. While initially effective in impeding tumor development in many glioma patients, chemoresistance often develops, leading to glioma recurrence. Mechanisms of resistance encompass DNA repair, alterations in cellular metabolism, and scavenging of ROS [38]. Several studies have indicated that BCAT1 overexpression is linked to multi-drug resistance, such as decreased chemosensitivity to CDDP in prostate cancer [39], and loss of BCAT1 overcoming resistance to EGFR tyrosine kinase inhibitors in lung cancer [40]. Nonetheless, the association of BCAT1 with adaptive response to temozolomide in glioma remains to be elucidated. In this study, we demonstrate that BCAT1 knockdown reduces intracellular GSH levels and increases oxidative stress. Critically, BCAT knockdown decreases IC50 values of temozolomide in glioma cells and BCAT1 overexpression confers resistance to temozolomide. Furthermore, we illustrate that U87 stable cells overexpressing CHIP exhibit a higher sensitivity to temozolomide. Mechanistically, GSH levels are significantly lower in U87-CHIP cells accompanied with elevated ROS levels. Additionally, Temozolomide treatment induces higher levels of oxidative stress in U87-CHIP cells and activates the intrinsic apoptosis pathway, resulting in more pronounced apoptotic cell death. Conversely, ectopic expression of BCAT1 elevates intracellular GSH levels and ameliorates oxidative stress, thereby increasing resistance to temozolomide both in vitro and in vivo. Overall, our findings suggest that BCAT1 may contribute to temozolomide resistance by facilitating GSH synthesis and ROS scavenging, while CHIP enhances sensitivity to temozolomide by promoting BCAT1 degradation.

In summary, our study elucidates the regulatory mechanisms of BCAT1 ubiquitination and degradation in glioma. We demonstrate that CHIP-mediated BCAT1 degradation inhibits glioma cell proliferation and enhances temozolomide sensitivity, thereby providing a potential therapeutic target for developing anti-tumor drugs.

## Supplementary information

Supplemental Figure 1-5

Supplemental Table 1

Raw western blot data

## Data Availability

The data that support the findings of this study are available from the corresponding author upon reasonable request.
