# Correction: CHIP-mediated ubiquitin degradation of BCAT1 regulates glioma cell proliferation and temozolomide sensitivity

**DOI:** 10.1038/s41419-026-08839-2

**Published:** 2026-05-19

**Authors:** Zhuo Lu, Xiao-Yu Wang, Kai-Yi He, Xin-Hao Han, Xing Wang, Zhen Zhang, Xin-Hui Qu, Zhi-Ping Chen, Xiao-Jian Han, Tao Wang

**Affiliations:** 1 Department of Thoracic Surgery, The First Affiliated Hospital, Jiangxi Medical College, Nanchang University, Nanchang, Jiangxi 330006 P.R. China; 2 Institute of Geriatrics, Jiangxi Provincial People’s Hospital, The First Affiliated Hospital of Nanchang Medical College, Nanchang, Jiangxi 330006 P.R. China; 3 Centre for Medical Research and Translation, Jiangxi Provincial People’s Hospital, The First Affiliated Hospital of Nanchang Medical College, Nanchang, Jiangxi 330006 P.R. China; 4 Institute of Clinical Medicine, Jiangxi Provincial People’s Hospital, The First Affiliated Hospital of Nanchang Medical College, Nanchang, Jiangxi 330006 P.R. China; 5 The Second Department of Neurology, Jiangxi Provincial People’s Hospital, The First Affiliated Hospital of Nanchang Medical College, Nanchang, Jiangxi 330006 P.R. China

**Keywords:** CNS cancer, Tumour-suppressor proteins, Ubiquitylation, Cell death

Correction to: *Cell Death & Disease* 10.1038/s41419-024-06938-6, published online 29 July 2024

The authors regret to inform that an error was present in Fig. 5E during figure layout, resulting in the representative image of siCHIP-2 group being a duplicate of siCHIP-1 group. All authors have reviewed and approved this correction. We apologize for the error and confirm that this correction does not alter the results or conclusion of the article.

**Original Figure 5** 
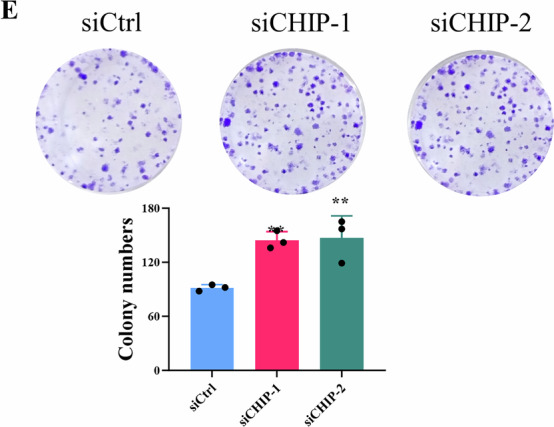


**Amended Figure 5** 
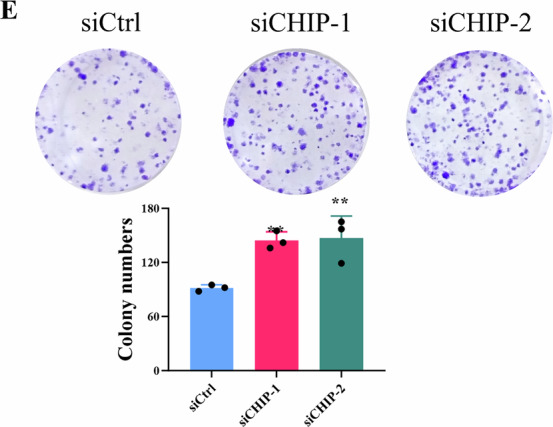


The original article has been corrected.

