# Supplementary material for: CHIP-mediated ubiquitin degradation of BCAT1 regulates glioma cell proliferation and temozolomide sensitivity
Source: Cell Death Dis. 2024 Jul 29;15(7):538. doi: 10.1038/s41419-024-06938-6 (PMC11286746; doi:10.1038/s41419-024-06938-6)
Supplement: Supplementary file 1 — Supplemental Figure 1-5 [file 41419_2024_6938_MOESM1_ESM.docx]

**Supplemental Figure 1**

**
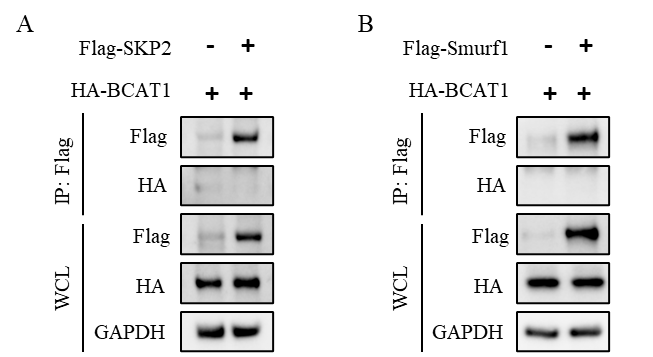
**

**Supplemental Figure 1. BACT1 has no interaction with SKP2 or Smurf1.** (A-B) U251 cells were transfected with HA-BCAT1 plasmid with or without Flag-SKP2 (A) or Flag-Smurf1 (B) plasmid. The interaction between BCAT1 and SKP2 or Smurf1 was detected by immunoprecipitation and western blot.

**Supplemental Figure 2**


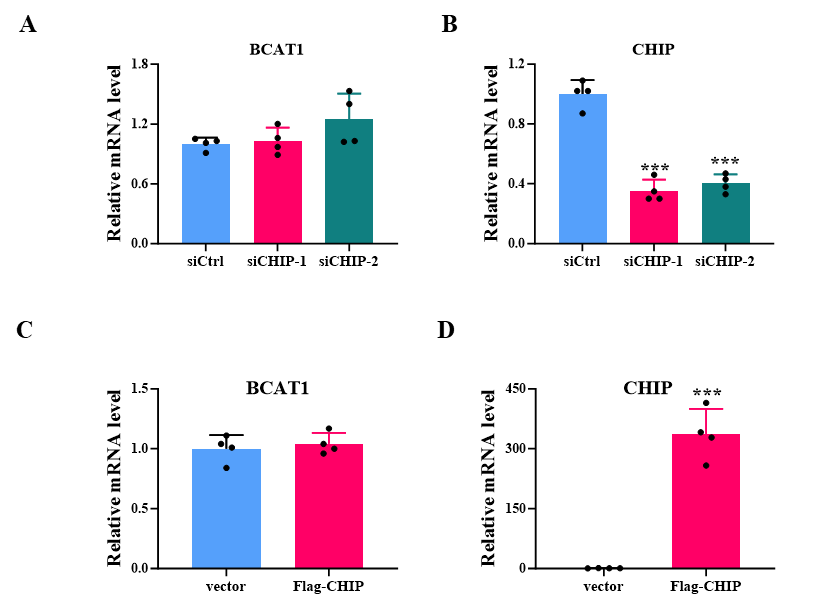


**Supplemental Figure 2. Effect of CHIP overexpression or knockdown on BCAT1 mRNA expression.** (A-B) U251 cells were transfected with scramble siRNA or CHIP siRNA. BCAT1 and CHIP mRNA levels were detected by RT-qPCR. (C-D) U251 cells were transfected with Flag-tagged CHIP plasmid. BCAT1 and CHIP mRNA levels were detected by RT-qPCR. Data were presented as mean ± SD. ****p* < 0.001.

**Supplemental Figure 3**


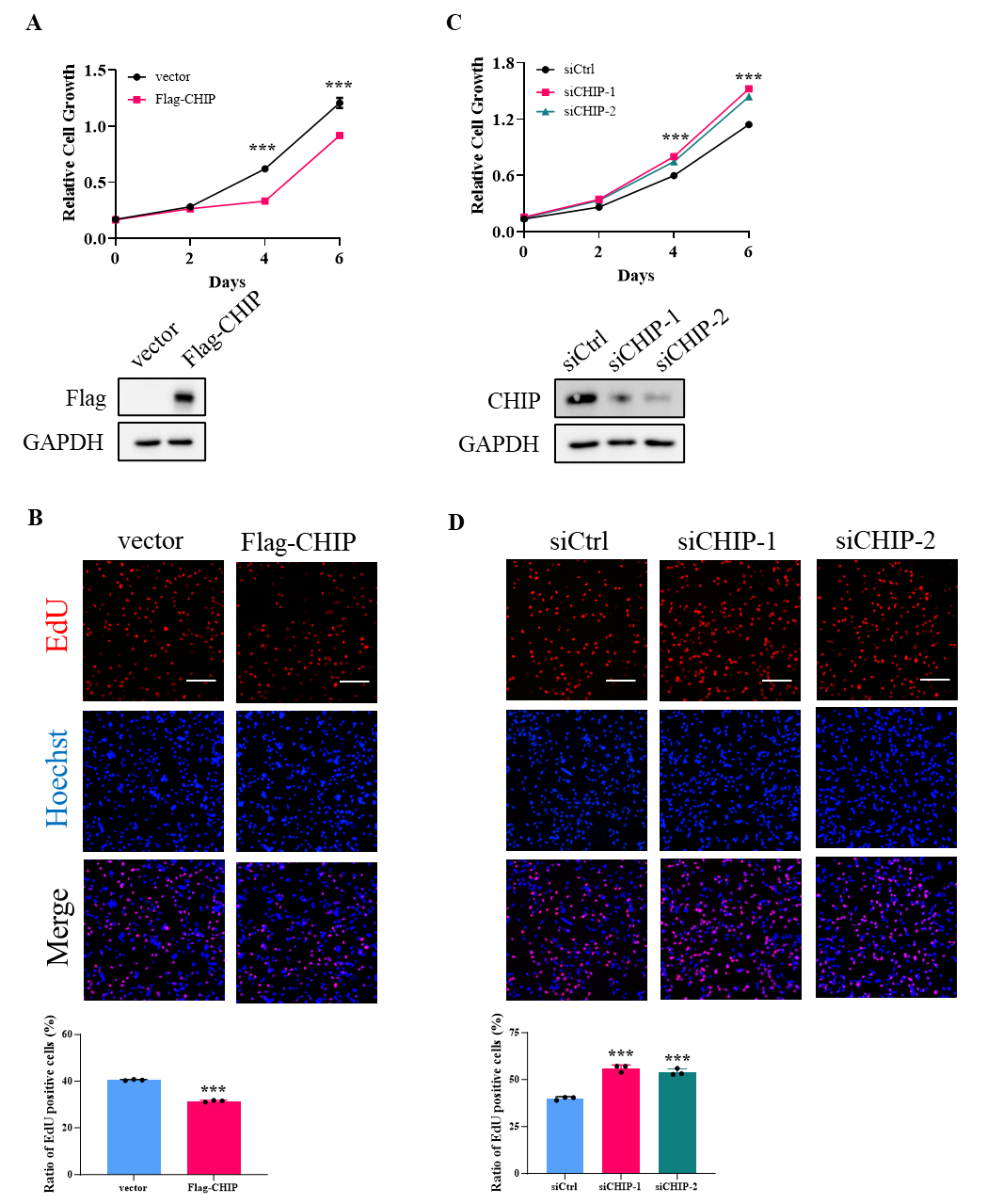


**Supplemental Figure 3. CHIP inhibits glioblastoma cell proliferation.** (A-B) U87 cells were transfected with vector or Flag-CHIP plasmid. Cell proliferation was detected by crystal violet staining and EdU staining. (C-D) U87 cells were transfected with scramble siRNA or CHIP siRNA. Cell proliferation were detected by crystal violet staining and EdU staining. Scale bar = 200 μm. Data were presented as mean ± SD of three independent experiments. ****p* < 0.001.

**Supplemental Figure 4**

**
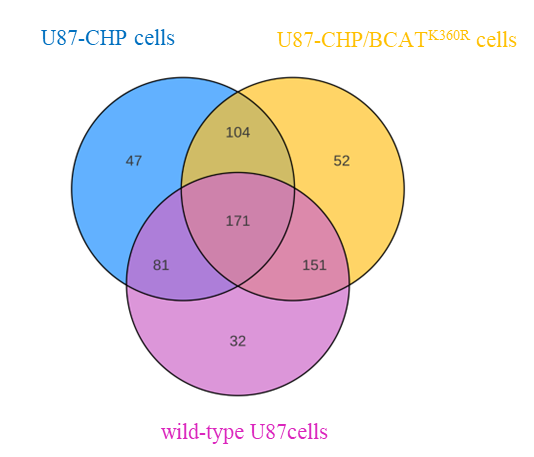
**

**Supplemental Figure 4. Venn diagram analysis of different metabolites.** Extracts from wild-type U87 cells, U87-CHIP and U87-CHIP/BCAT1^K360R^ cells were analyzed using an LC-ESI-MS/MS system.

**Supplemental Figure 5**


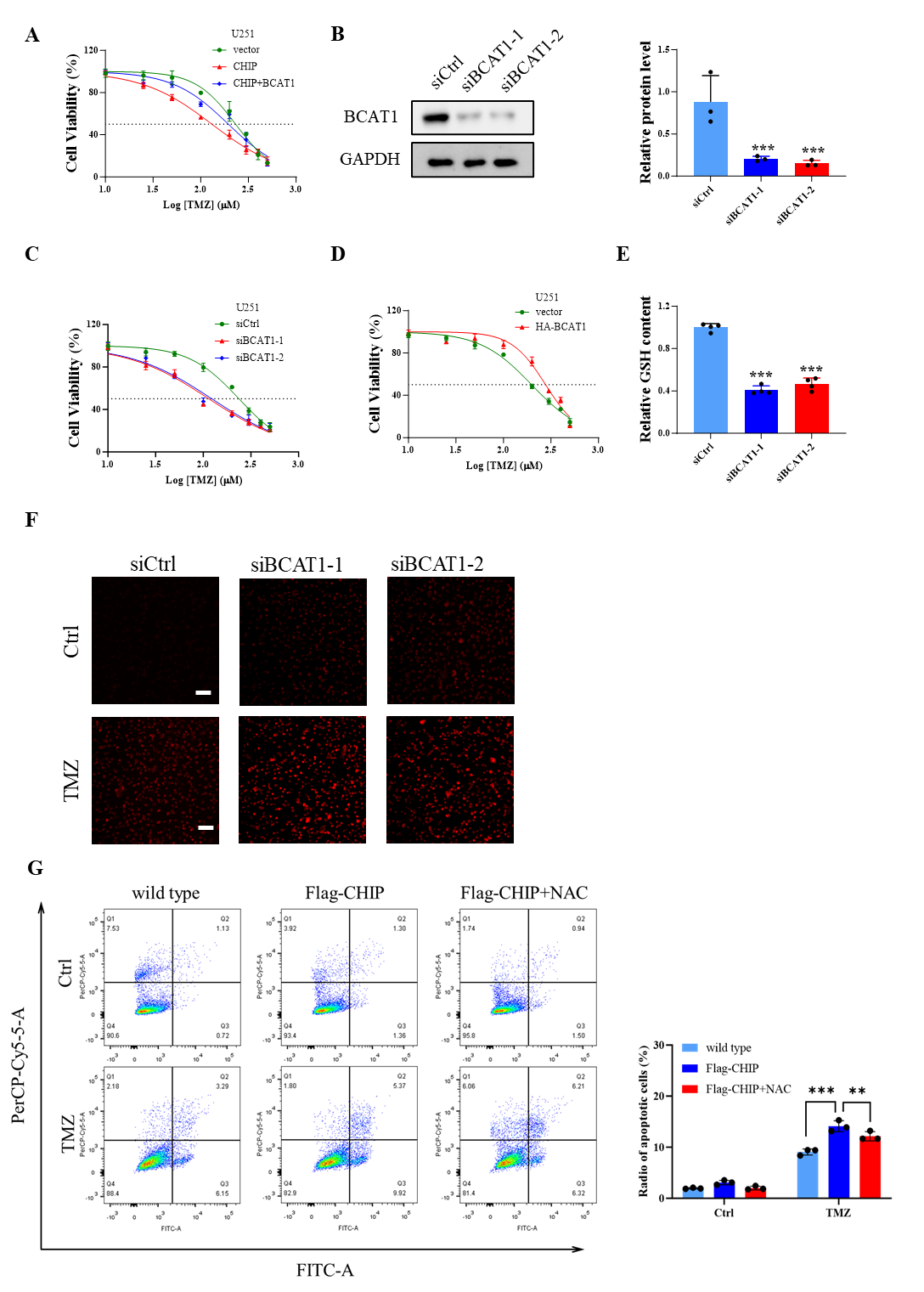


**Supplemental Figure 5. BCAT1 knockdown sensitize U251 cells to temozolomide.** (A) U251 cells were transfected with Flag-CHIP plasmid with or without HA-BCAT1 plasmid, and treated with various concentrations of temozolomide for 48 h. Cell viability was detected by CCK8 assay. (B) U251 cells were transfected with scramble siRNA or BCAT1 siRNA. BCAT1 expression was detected by western blot. (C) U251 cells were transfected with scramble siRNA or BCAT1 siRNA, and treated with different concentrations of temozolomide for 48 h. Cell viability was detected by CCK-8 assay kit. (D) U251 cells were transfected with vector or HA-BCAT1 plasmid, and treated with various concentrations of temozolomide for 48 h. Cell viability was detected by CCK8 assay. (E) U251 cells were transfected with scramble siRNA or BCAT1 siRNA. Intracellular GSH levels were detected. (F) U251 cells were transfected with scramble siRNA or BCAT1 siRNA, and treated with 100 μM temozolomide for 48 h. Intracellular ROS level was detected by DHE staining. Scale bar = 100 μm. (G) Wild type U87 cells and U87-CHP cells were treated with 100 μM temozolomide in the presence or absence of 5 mM NAC for 48 h. The ratio of apoptotic cells were detected by Annexin/PI staining and flow cytometry. Data were presented as mean ± SD. ***p* < 0.01, ****p* < 0.001.
