# Supplementary material for: CHIP-mediated ubiquitin degradation of BCAT1 regulates glioma cell proliferation and temozolomide sensitivity
Source: Cell Death Dis. 2024 Jul 29;15(7):538. doi: 10.1038/s41419-024-06938-6 (PMC11286746; doi:10.1038/s41419-024-06938-6)
Supplement: Supplementary file 2 — Supplemental Table 1 [file 41419_2024_6938_MOESM2_ESM.docx]

**Supplemental Table 1**. **Primers used in this study**

| **Construction of recombinant plasmid** | |
| --- | --- |
| CHIP-F | 5’-GATAGATCTGATATCGGTACCAATGAAGGGCAAGGAGGAGAAG-3’ |
| CHIP-R | 5’-CCTCTAGAGTCGACTGGTACCTCAGTAGTCCTCCACCCAGC-3’ |
| CHIP-K30A-F | 5’-AGCGCGCAGGAGCTCGCGGAGCAGGGCAATCGT-3’ |
| CHIP-K30A-R | 5’-ACGATTGCCCTGCTCCGCGAGCTCCTGCGCGCT-3’ |
| CHIP-H260Q-F | 5’-AAGGACATCGAGGAGCAGCTGCAGCGTGTGGGT-3’ |
| CHIP-H260Q-R | 5’-ACCCACACGCTGCAGCTGCTCCTCGATGTCCTT-3’ |
| BCAT1-pLV-F | 5’-TATAGGGCCCGGGTTGGATCCGCCACCATGAAGGATTGCAGTAACGG-3’ |
| BCAT1-pLV-R | 5’-AGGCCGGAGACGCGTGGATCCTCAGGATAGCACAATTGTCC-3’ |
| BCAT1-F | 5’-ACCGAGATCTCTCGAGGTACCATGAAGGATTGCAGTAACGG-3’ |
| BCAT1-R | 5’-GATCCCCGCGGCCGCGGTACCTCAGGATAGCACAATTGTCC-3’ |
| BCAT1-K19R-F | 5’-GGAGAAGGAGGATCACGAGAGGTGGTGGGGACT-3’ |
| BCAT1-K19R-R | 5’-AGTCCCCACCACCTCTCGTGATCCTCCTTCTCC-3’ |
| BCAT1-K28R-F | 5’-GGGACTTTTAAGGCTCGAGACCTAATAGTCACA-3’ |
| BCAT1-K28R-R | 5’-TGTGACTATTAGGTCTCGAGCCTTAAAAGTCCC-3’ |
| BCAT1-K107R-F | 5’-CGAGGAGTAGATAATCGAATTCGACTGTTTCAG-3’ |
| BCAT1-K107R-R | 5’-CTGAAACAGTCGAATTCGATTATCTACTCCTCG-3’ |
| BCAT1-K360R-F | 5’-ATGGAGAATGGTCCTCGGCTGGCAAGCCGCATC-3’ |
| BCAT1-K360R-R | 5’-GATGCGGCTTGCCAGCCGAGGACCATTCTCCAT-3’ |
| BCAT1-K368R-F | 5’-AGCCGCATCTTGAGCCGATTAACTGATATCCAG-3’ |
| BCAT1-K368R-R | 5’-CTGGATATCAGTTAATCGGCTCAAGATGCGGCT-3’ |
| **Quantitative RT-PCR** | |
| BCAT1-qPCR-F | 5’-CGGAGAAGGAGGATCAAAAGAGGT-3’ |
| BCAT1-qPCR-R | 5’-ATGAGCCAGGGTGCAATGACAGGT-3’ |
| CHIP-qPCR-F | 5’-CCGACTACCTGTGTGGCAAGAT-3’ |
| CHIP-qPCR-R | 5’-CAGAGATGAATGCGTCAATAAC-3’ |
| GAPDH-qPCR-F | 5’-ACAACTTTGGTATCGTGGAAGG-3’ |
| GAPDH-qPCR-R | 5’-GCCATCACGCCACAGTTTC-3’ |
