# Supplementary material for: CHIP-mediated ubiquitin degradation of BCAT1 regulates glioma cell proliferation and temozolomide sensitivity
Source: Cell Death Dis. 2024 Jul 29;15(7):538. doi: 10.1038/s41419-024-06938-6 (PMC11286746; doi:10.1038/s41419-024-06938-6)

Figure 1

A

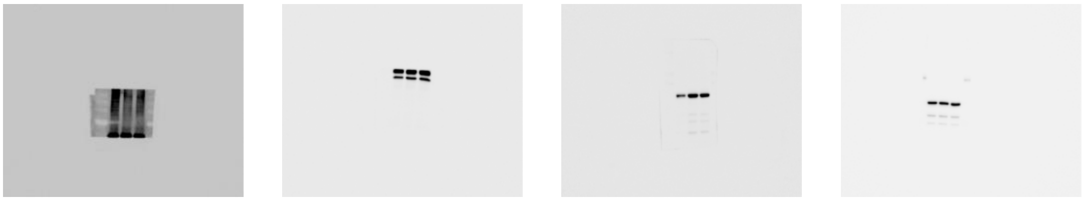

B

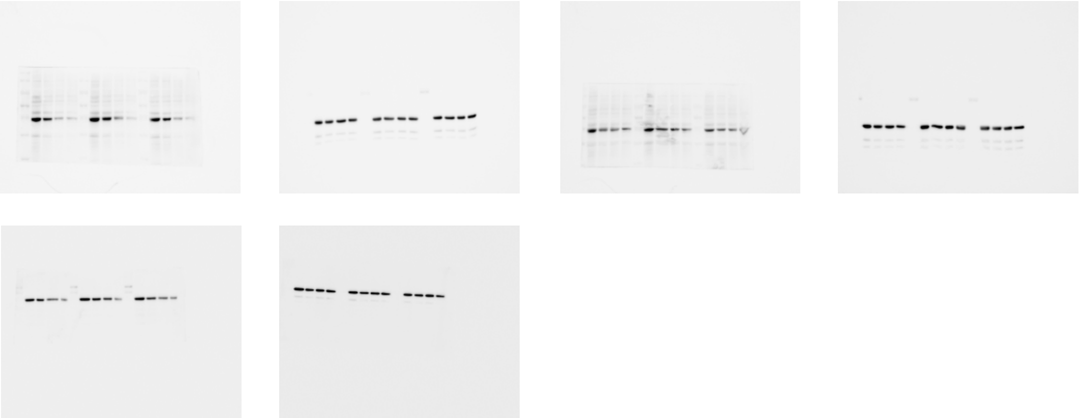

C

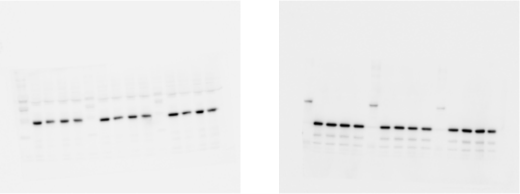

D

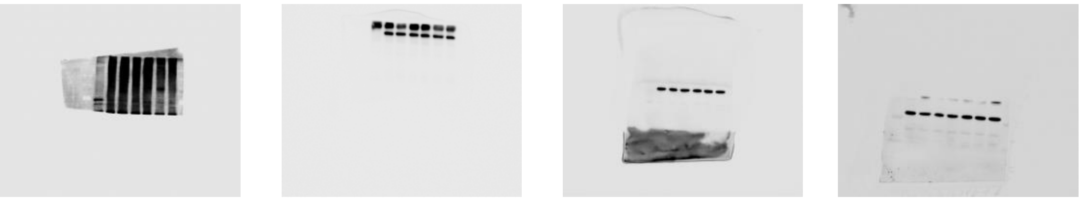

E

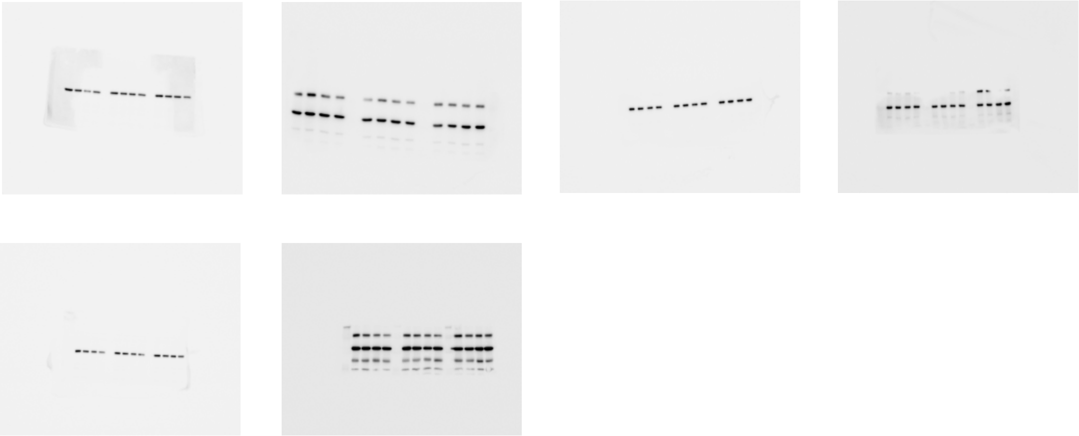

Figure 2

B

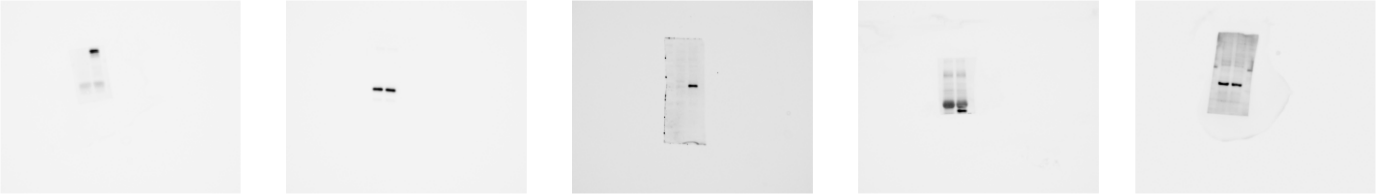

C

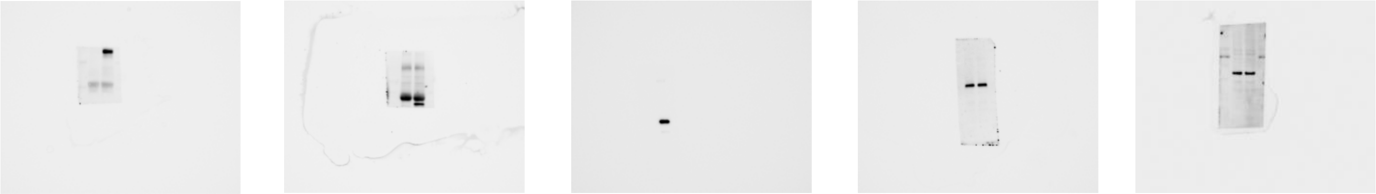

E

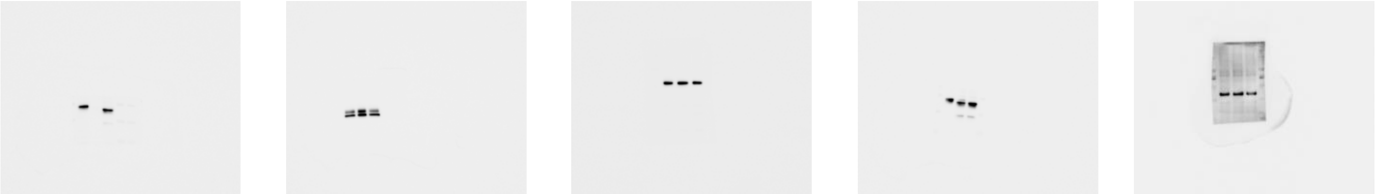

F

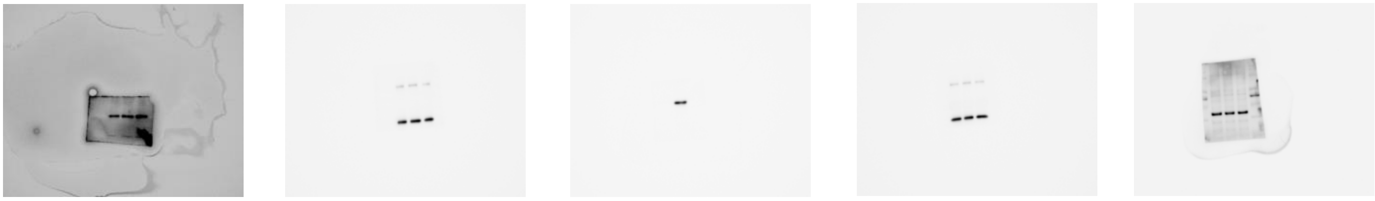

G

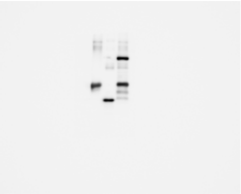

Figure 3

A

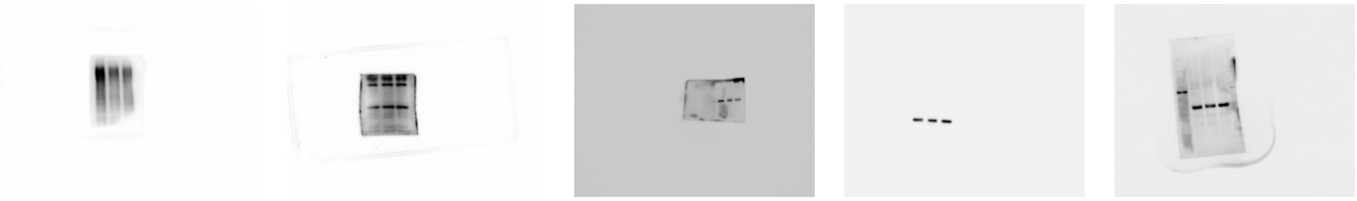

B

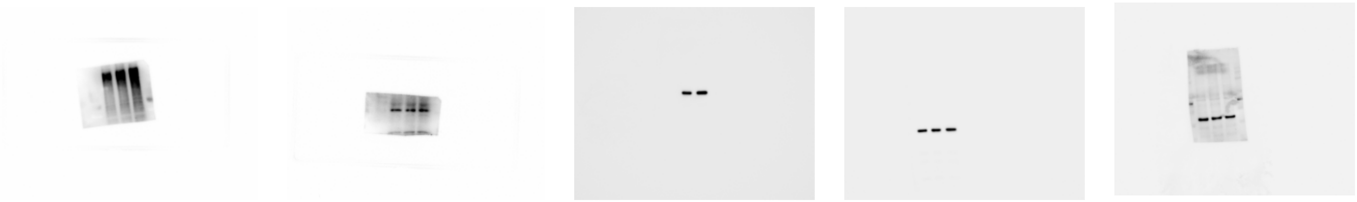

C

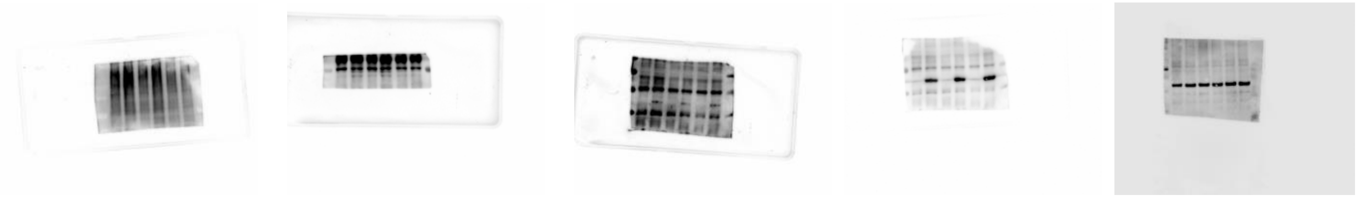

D

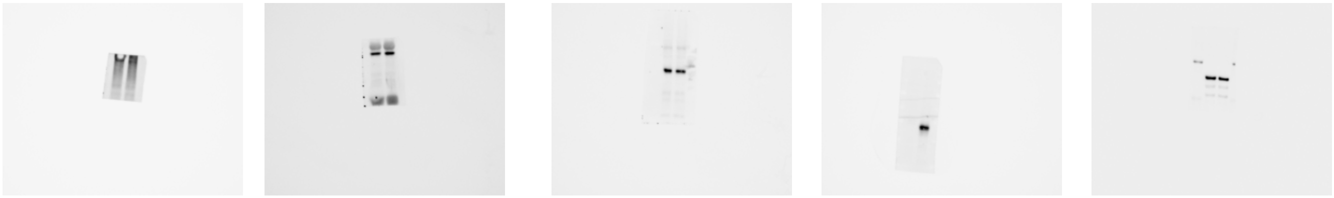

E

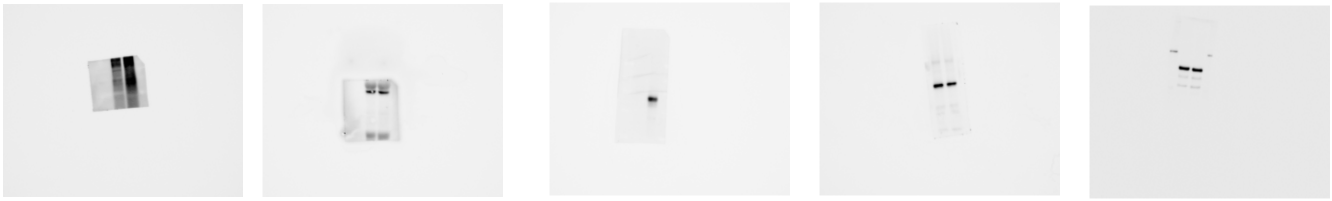

F

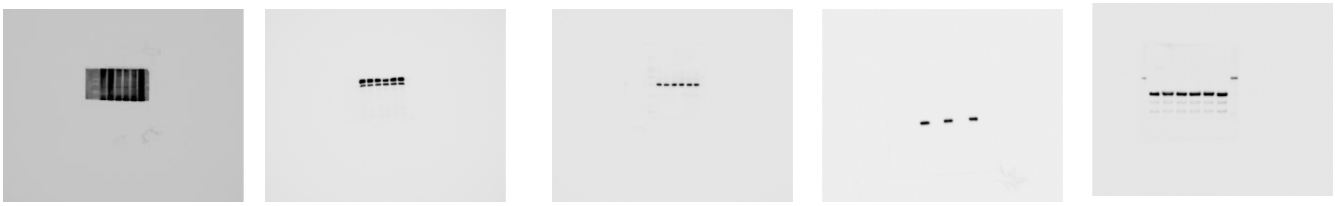

G

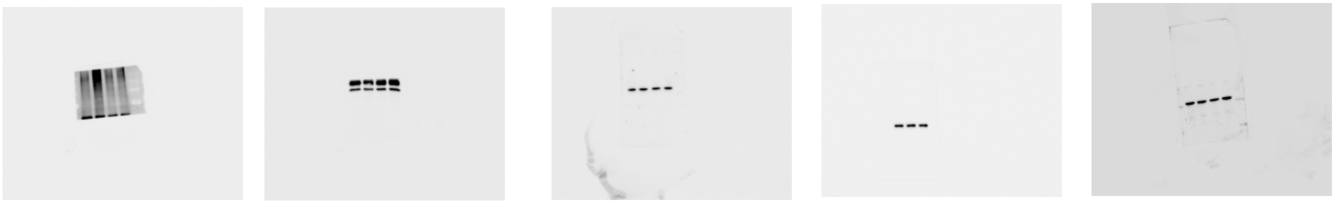

Figure 4

A

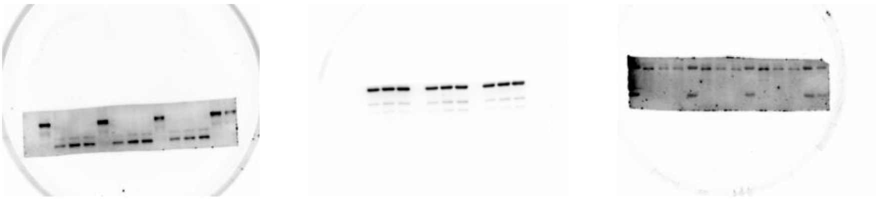

B

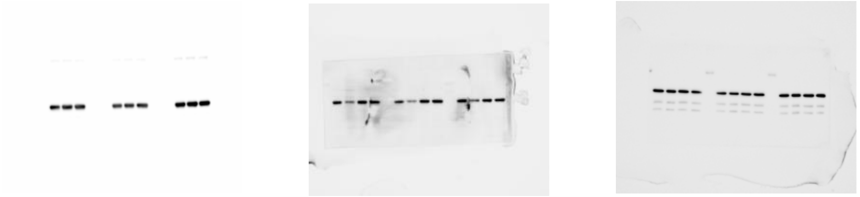

C

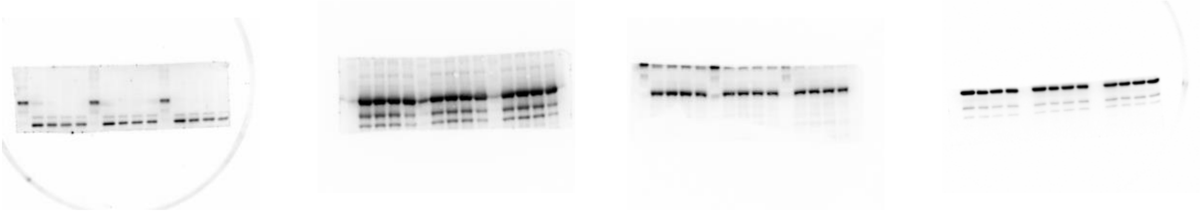

D

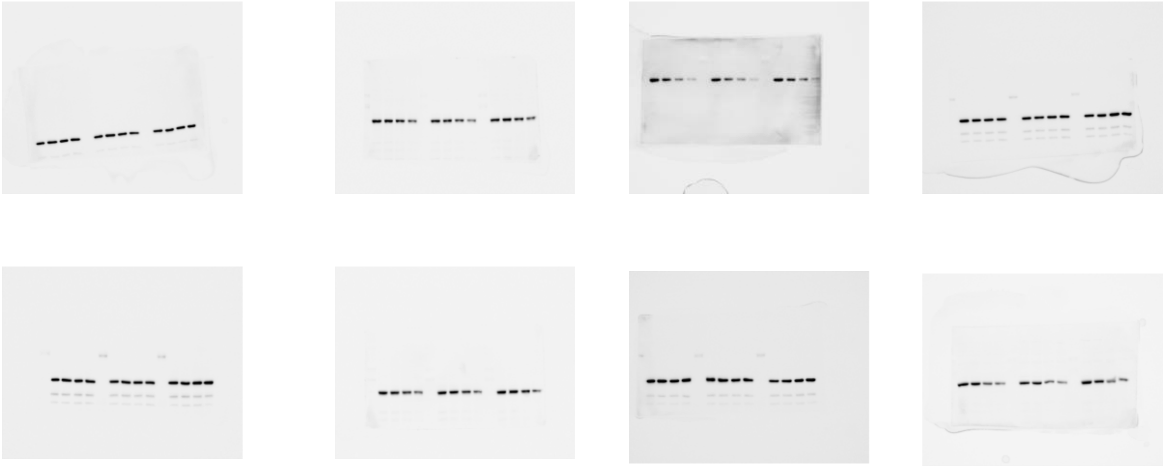

E

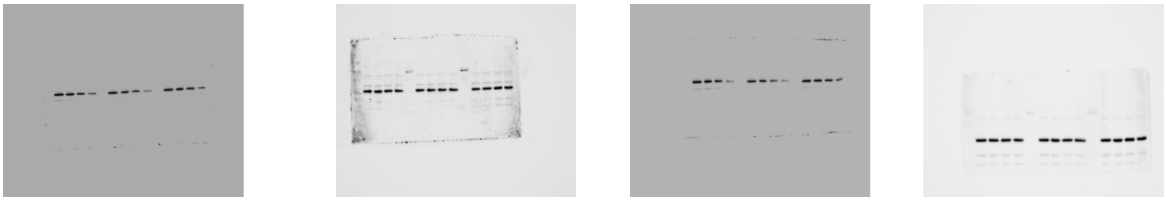

Figure 5

A

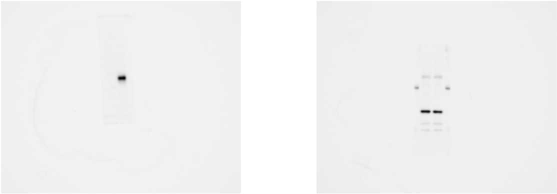

D

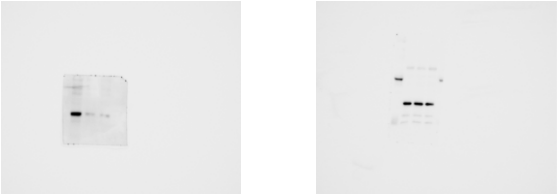

Figure 6

A

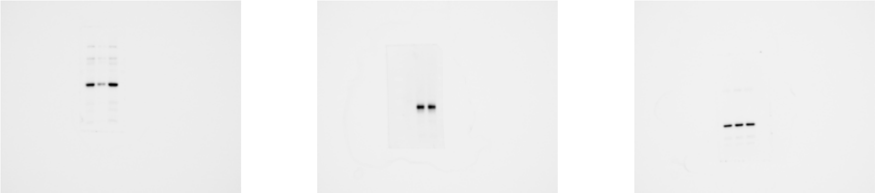

Figure 9

G

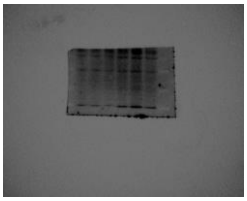

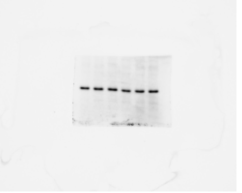

Figure S1

A

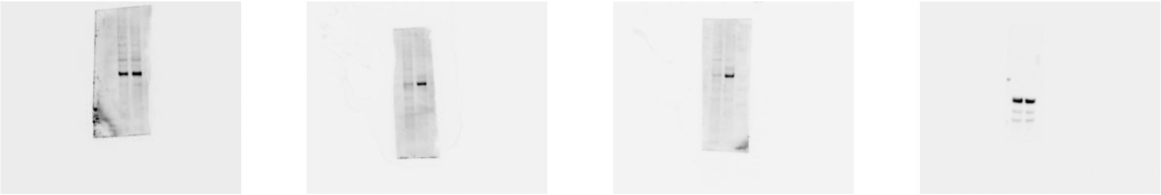

B

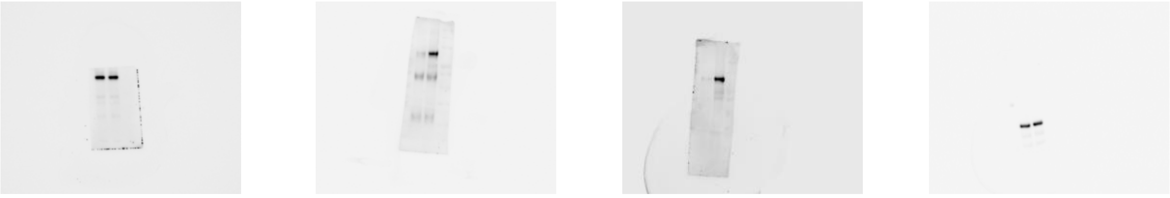

Figure S3

A

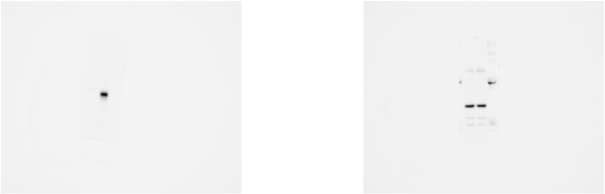

C

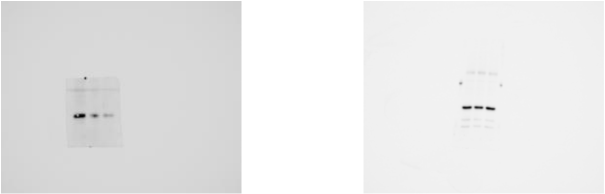

Figure S5

B

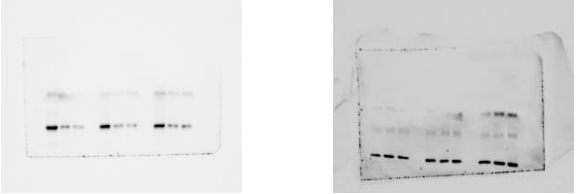

Supplement: Supplementary file 3 — Raw western blot data [file 41419_2024_6938_MOESM3_ESM.pdf]
